# Supplementary material for: Molecular properties and intramolecular interactions of peptide-conjugated phosphorodiamidate morpholino oligonucleotides
Source: Mol Ther Nucleic Acids. 2025 Aug 14;36(3):102685. doi: 10.1016/j.omtn.2025.102685 (PMC12408397; doi:10.1016/j.omtn.2025.102685)
Supplement: Document S1. Figures S1–S5, Tables S1 and S2, and Supplemental Methods [file mmc1.pdf]

OMTN, Volume *36*

Supplemental information

Molecular properties and intramolecular interactions of peptide-conjugated phosphorodiamidate morpholino oligonucleotides

Evgenii Kliuchnikov, Farkhad Maksudov, Daniel Pierson, Kenneth A. Marx, Arani Chanda, and Valeri Barsegov

Supplemental Methods

Force field development for all-atom simulations of PPMO molecules: In our previous study [1], we derived the atomic partial charges and force-field parameters for the following three parts of the PMO structure: i) for the phosphorodiamidate group attached to the 5′-end and capping the HN atomic group attached to the N3′ atom (mimicking the PMO 5′-end); ii) for the phosphorodiamidate group attached to the 3′-end and capping the HO atomic group attached to the O5′ atom (mimicking the PMO 3′-end); and iii) for the phosphorodiamidate group attached to both 5′- and 3′-ends. In the same study [1], we also derived the force field parameters and partial charges for the morpholino triethylene glycol (MTEG) linker attached to the 5′-end of PPMO through the phosphorodiamidate group. For the peptide portion of PPMO, we used the original Amber ff14SB force field for amino acids, since the peptide did not have any chemical modifications. In the derivation of partial charges in the PMO part of the PPMO structure carried out in our previous study [1], we employed the Restrained Electro Static Potential (PESP) charge fitting procedure [2], implemented in the RED server (RESP ESP charge Derive Server) [3]. We utilized the RESP method in conjunction with HF theory and the 6-31G* basis set. Several *ab initio* calculations were carried out for each fragment, including the morpholino ring (5′-, 3′-, and central) with guanine, cytosine, thymine, and adenine bases, and with the MTEG linker. For each fragment we carried out 3 runs (total of 39 runs) over which the final partial charges were averaged. The Antechamber package [4] was employed to assign the all-atom types for atoms in these fragments. Molecular mechanical parameters were obtained from the bsc0$_{\chi OL3}$ force field for nucleic acids and from the ff14SB force field for proteins, for the included atom types. These include the equilibrium bond distances (r_0), spring constants for covalent bonds (k_b), equilibrium bond angles (θ_0), and spring constants for bending of bond angles (k_θ), as well as torsional angle parameters (magnitude associated with torsion energy ($V_n/2$), phase offset (γ) and periodicity (n)). For the atom types not covered in the bsc0$_{\chi OL3}$ and ff14SB force fields, the molecular mechanical parameters were generated using the general Amber force field GAFF [5]. For model detail of the force field development procedure, including the molecular mechanics parameters and partial charges, the reader should consult our previous work [1] (see also the SI in Ref. [1]). For each PPMO molecule, we created the topology files for each of the "morpholino nucleotides", for the MTEG linker, and for the peptide part. Next, we linked "morpholino nucleotides" through the phosphorodiamidate groups, added the MTEG linker at the 5′-end, and linked the poly-Arg peptide to the 3′-end of each of the target sequences (see Fig. 1 in the main part). These structures were used in all the MD simulations described in the main part.

Reference structures for calculation of thermodynamic state functions: To generate the unfolded reference structures for the 22-mer, 25-mer, and 30-mer PPMO molecules, we performed the all-atom MD simulations of thermal unfolding using the Generalized Born (GB) model of implicit solvation [6] implemented in pmemd [7]. We used the solution conformations obtained from the numerical fit of the theoretical CD profiles to the experimental CD curves. Each of the PPMO molecules were gradually heated from 300 K to 500 K over a 2.5-μs time interval to unfold (see Fig. S2). Next, the Support Vector Classifier was used to identify the 'unfolded' conformations (Fig. S2). We selected a total of 62, 98, and 84 extended conformations for the 22-mer, 25-mer, and 30-mer PPMO, respectively. Using these structures, the average reference enthalpy (H_{ref}) and reference entropy (S_{ref}) were then generated for the extended initial conformations for the 22-mer, 25-mer, and 30-mer PPMOs displayed in Fig. 1B in the main part.

Classification of folded and unfolded conformations of PPMOs: We applied the Support Vector Machines (SVM) to perform classification of the 22-mer, 25-mer, and 30-mer PPMO conformations into

the folded and unfolded conformation classes. We employed We carried out 4 independent 2.5-μs long thermal unfolding MD simulations to find the unfolded conformations for the 22-mer, 25-mer, and 30-mer PPMO molecules. The first 4 most populated conformations for each PPMO were used as initial structures (first 5 of them are shown in Fig. 2 in the main part). During these simulations, each PPMO was gradually heated from 300 K to 500 K. The numerical output (coordinate and energy files) was then used to identify the most representative molecular properties that show the unfolding transitions in the 22-mer, 25-mer, and 30-mer PPMO molecules at high temperatures. *Order parameters:* The temperature-dependent profiles of the end-to-end distance X, radius of gyration R_g, number of base stacks N_{BS}, and Solvent Accessible Surface Area $SASA$ showed the sigmoidal shape characteristic of a phase transition (Fig. S2). This enabled us to select the unfolded conformations of the 22-mer, 25-mer, and 30-mer PPMOs in order to form the training set for the Machine Learning based classification of the ensemble of conformations for these PPMOs. We selected the conformations for which the values of X, R_g, N_{BS}, and $SASA$ fall within the upper 90% of their maximum values, as indicated by the horizontal dashed lines in Figs. S2A-B.

For each PPMO system, we constructed the dataset with the values of X, R_g, N_{BS}, $SASA$ and $eRMSD$. For a manifold of various conformations of the PPMO molecules, obtained from the equilibrium MD simulations in an aqueous solution at 300 K, we constructed datasets $D_{eq,22}$, $D_{eq,25}$ and $D_{eq,30}$ for the 22-mer, 25-mer, and 30-mer PPMOs, respectively. The numerical output data for PPMOs' conformations, obtained from the MD simulations of thermal unfolding, were used to construct datasets $D_{unf,22}$, $D_{unf,25}$ and $D_{unf,30}$ for the 22-mer, 25-mer, and 30-mer PPMOs, respectively. For each PPMO system, these datasets were combined into datasets D_{22}, D_{25} and D_{30}, with two classes labeled 'Folded' and 'Unfolded'. These combined datasets were randomly separated into two halves forming the training sets ($D_{train,22}$, $D_{train,25}$ and $D_{train,30}$) and the test sets ($D_{test,22}$, $D_{test,25}$ and $D_{test,30}$). Next, we employed SVM to perform data classification, i.e. separation of PPMOs' structures into the folded conformations ('Folded' class) and the unfolded conformations ('Unfolded' class). The implementation of the SVM algorithm was based on the Python scikit-learn package [8]. Since the test sets contain only one half of the unfolded structures generated at 300 K, next, we applied these pre-trained SVM models to the data sets $D_{eq,22}$, $D_{eq,25}$ and $D_{eq,30}$, in order to extract all the unfolded PPMOs' conformations that would exclude conformations from the MD simulations of thermal unfolding. Therefore, we identified 62 conformations for the 22-mer PPMO, 98 conformations for the 25-mer PPMO, and 84 conformations for the 30-mer PPMO. The classification results for the 30-mer PPMO conformations into the 'Folded' class and 'Unfolded' class are displayed in Fig. S2 showing two- and three-dimensional projections of the separating hypersurface, i.e. N_{BS} vs. R_g (Fig. S2C) and N_{BS} vs. $SASA$ (Fig. S2D) and X vs. R_g vs. $SASA$ (Fig. S2E).

Supplemental Table

Table S1. Molecular properties and energetic characteristics of solution conformations of 22-mer PPMO, 25-mer PPMO, 30-mer PPMO: Shown for each principal solution conformer i = I-III, which account for ~55-70% of the ensemble populations, are the equilibrium population w_i, end-to-end distance X_i, radius of gyration $R_{g,i}$, number of base pairs $N_{BP,i}$, numbers of base stacks $N_{BS,i}$, solvent accessible surface area ($SASA_i$), and intrinsic viscosity η_i. Also shown are changes in free energy ΔG_i and enthalpy ΔH_i for folding of PPMOs (at T = 300 K). Data for X_i, $R_{g,i}$, $SASA_i$, ΔG_i, and ΔH_i for PPMOs (bolded text) are compared with the contributions to these quantities from the PMO part (separated by the slash).

**22-mer PPMO** / PMO in PPMO								
	w_i	X_i, nm	$R_{g,i}$, nm	$N_{BP,i}$/ $N_{BS,i}$	$SASA_i$, Å^2	η_i, cm^3/g	ΔG_i, kcal/mol	ΔH_i, kcal/mol
I	**0.26**	**1.9**/1.0	**1.3**/1.3	**9/4**	**4791**/3613	**5.3**	**-58**/-45	**-93**/-87
II	**0.17**	**1.7**/1.7	**1.4**/1.4	**7/5**	**5314**/3967	**4.5**	**-42**/-23	**-57**/-52
III	**0.09**	**3.2**/3.2	**1.4**/1.4	**2/5**	**5110**/3855	**4.5**	**-57**/-16	**-82**/-46
**25-mer PPMO** / PMO in PPMO								
	w_i	X_i, Nm	$R_{g,i}$, Nm	$N_{BP,i}$/ $N_{BS,i}$	$SASA_i$, Å^2	η_i, cm^3/g	ΔG, kcal/mol	ΔH, kcal/mol
I	**0.30**	**1.3**/1.7	**1.5**/1.4	**5/8**	**5215**/4632	**6.3**	**-83**/-72	**-118**/-90
II	**0.25**	**1.6**/1.8	**1.4**/1.4	**5/10**	**5763**/4820	**5.0**	**-65**/-58	**-97**/-77
III	**0.12**	**2.3**/1.8	**1.3**/1.3	**2/7**	**5486**/4911	**4.2**	**-79**/-48	**-101**/-66
**30-mer PPMO** / PMO in PPMO								
	w_i	X_i, nm	$R_{g,i}$, Nm	$N_{BP,i}$/ $N_{BS,i}$	$SASA_i$, Å^2	η_i, cm^3/g	ΔG, kcal/mol	ΔH, kcal/mol
I	**0.27**	**2.3**/1.3	**1.5**/1.5	**5/10**	**6506**/5596	**6.1**	**-84**/-44	**-104**/-71
II	**0.23**	**2.2**/2.5	**1.4**/1.4	**6/12**	**6179**/5057	**4.3**	**-80**/-37	**-114**/-68
III	**0.22**	**2.4**/3.4	**1.4**/1.4	**3/10**	**6276**/5406	**4.7**	**-60**/-18	**-110**/-52

Table S2. Molecular properties and thermodynamic state functions for the three therapeutic 22-mer, 25-mer, and 30-mer PPMOs: Presented are the statistics (averages and standard deviations) of the radius of gyration R_g, number of base pairs N_{BP}, number of base stackings N_{BS}, solvent accessible surface area $SASA$, intrinsic viscosity $[\eta]$, and Huggins constant k_H. Also shown are the changes in free energy ΔG and enthalpy ΔH for folding for the average PPMO solution structures (at $T = 300$ K); these quantities were calculated using the PPMOs' unfolded structures as the reference states (Fig. 1B) and the 6-7 most populated structures. Data for the PPMO structures are compared with data for the PMO structures from our previous study [1] (separated by the slash). The largest and the smallest parameter values are given in red and light blue color.

Parameter	22-mer PPMO / 22-mer PMO	25-mer PPMO / 25-mer PMO	30-mer PPMO / 30-mer PMO
R_g, nm	1.5±0.2 / 1.5±0.2	1.4±0.1 / 1.4±0.1	1.5±0.2 / 1.7±0.1
N_{BP}	6.0±2.1 / 3.3±0.9	4.9±2.9 / 5.9±1.1	4.5±2.2 / 6.4±4.2
N_{BS}	5.1±1.7 / 6.5±3.6	8.4±2.8 / 8.3±3.2	10.9±2.4 / 8.7±3.0
$SASA$, Å^2	5247±321 / 4538±248	5339±339 / 4819±158	6555±361 / 5945±341
$[\eta]$, cm^3/g	4.5±0.6 / 4.5±0.6	4.6±0.5 / 4.7±0.6	6.1±0.6 / 6.2±0.8
k_H	4.5 / 4.5	9.7 / 9.9	3.7 / 3.8
ΔG, kcal/mol	-49±9 / -34±15	-71±11 / -51±15	-70±23 / -50±10
ΔH, kcal/mol	-86±21 / -57±31	-103±22 / -89±19	-103±37 / -103±21

Supplemental Figures

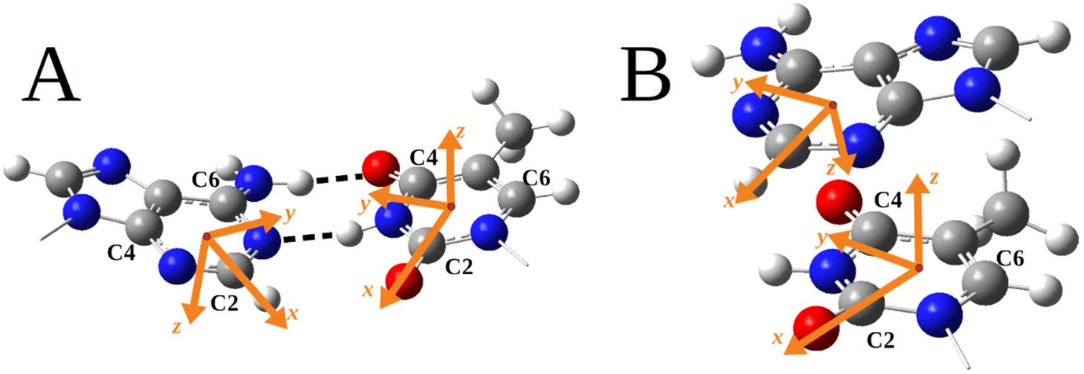

Figure S1. Base pairing and base stacking for PPMO molecules: Panel **A**: Local coordinate systems for purines and pyrimidines used in the calculation of base pairing and base stacking interactions (see Supplemental Methods). The center of the base ring atoms C2, C4, and C6 represents the origin of the coordinate system as shown in the graph. The x-and y-axes lie in the plane of the base while the z-axis is normal to the xy-plane. The x-axis is pointed in the C2-atom direction, and the y-axis is pointed toward the C4-atom (for C and U) or toward the C6-atom (for A and G). Two bases are forming a base pair via hydrogen bonds shown as dashed black lines. Panel **B**: The same x-, y- and z-axes from Panel **A** were used to describe the formation of base stacking interaction.

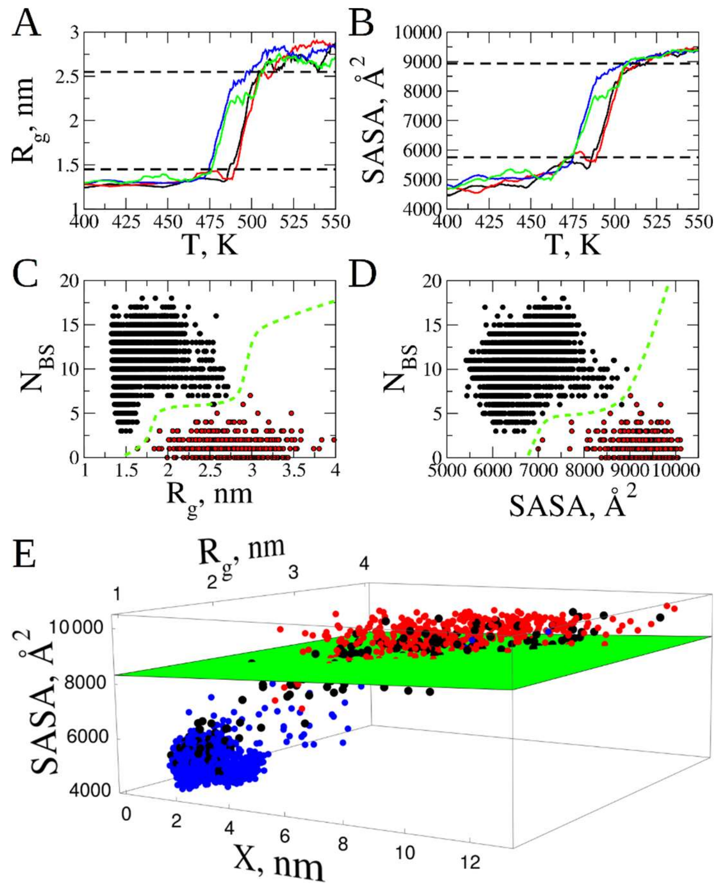

Figure S2. Dynamic structural properties associated with thermal unfolding of 30-mer PPMO: Panels **A-B** display temperature-dependent profiles of the secondary and tertiary structural properties of the 30-mer from three independent 2.5-μs MD simulations of gradual heating of this PPMO from 300 K to 500 K: radius of gyration R_g (panel **A**) and $SASA$ (panel **B**). Only the results from the 400 K – 500 K portion of the thermal unfolding experiments are shown. Horizontal dashed-dotted lines separate the regions corresponding to the folded and unfolded conformations of PPMOs. Also shown are the 2D scatter plots of a number of base-stacking interactions N_{BS} vs. R_g (panel **C**) and vs. $SASA$ (panel **D**) for the 30-mer PPMO, and 3D scatter plot of end-to-end distance X vs. R_g vs. $SASA$ (panel **E**). The black data points correspond to the 30-mer PPMO's conformations extracted from the equilibrium MD simulations at 300 K, while the red data points represent the conformations observed in the simulations of thermal unfolding, which were classified as 'Unfolded', and blue data points represent the conformations observed in the MD simulations of thermal unfolding, which were classified as 'Folded' (panel **E** only). In panels **C**, **D**, and **E**, the green dashed lines (plane) represent the two-dimensional (three-dimensional) projection of an optimal hypersurface obtained using the Support Vector Classifier to separate the conformations that belong to the 'Folded' class and 'Unfolded' class. In panels **C** and **D**, the black data points under the decision boundary line, which belong to the 'Unfolded' class, are the initial unfolded (reference) structures used in data analysis (see Fig. 1B in the main text). In panel **E**, the black data points above the hyperplane, which belong to the 'Unfolded' class, are also the initial unfolded (reference) structures used in data analysis (see Fig. 1B in the main text).

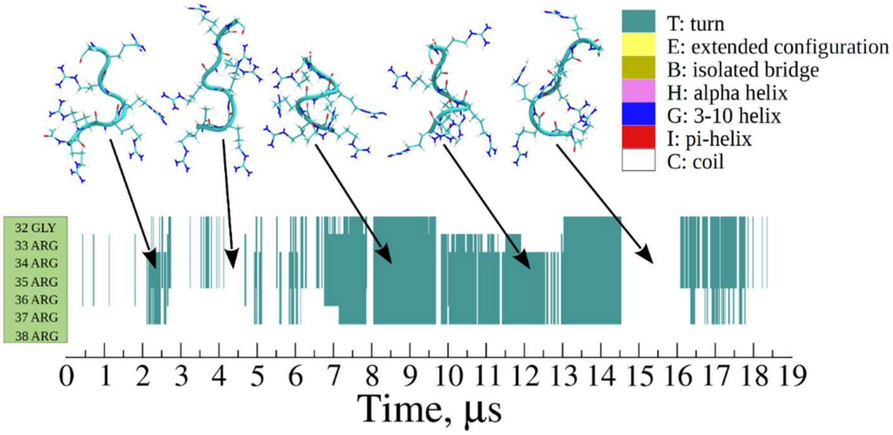

Figure S3. Secondary structure propensity for the peptide portion of the 22-mer, 25-mer, and 30-mer PPMOs: Displayed are the dynamic changes in the secondary structure for the peptide portion in the 30-mer PPMO from the 19-µs Molecular Dynamics simulations (all 15 trajectories combined in one long run). Color denotation for various secondary structure elements (i.e. α-helix, 3_{10}-helix, π-helix, β-strand, random coil, extended state, turn, and bridge) is shown in the graph. Also shown are several structure snapshots corresponding to 5 timepoints: 2.5 µs, 4.5 µs, 8.6 µs, 12.2 µs, and 15.5 µs. Only random coil and turn are observed during 19 µs of conformational dynamics (similar results were obtained for the 22-mer and 25-mer PPMOs).

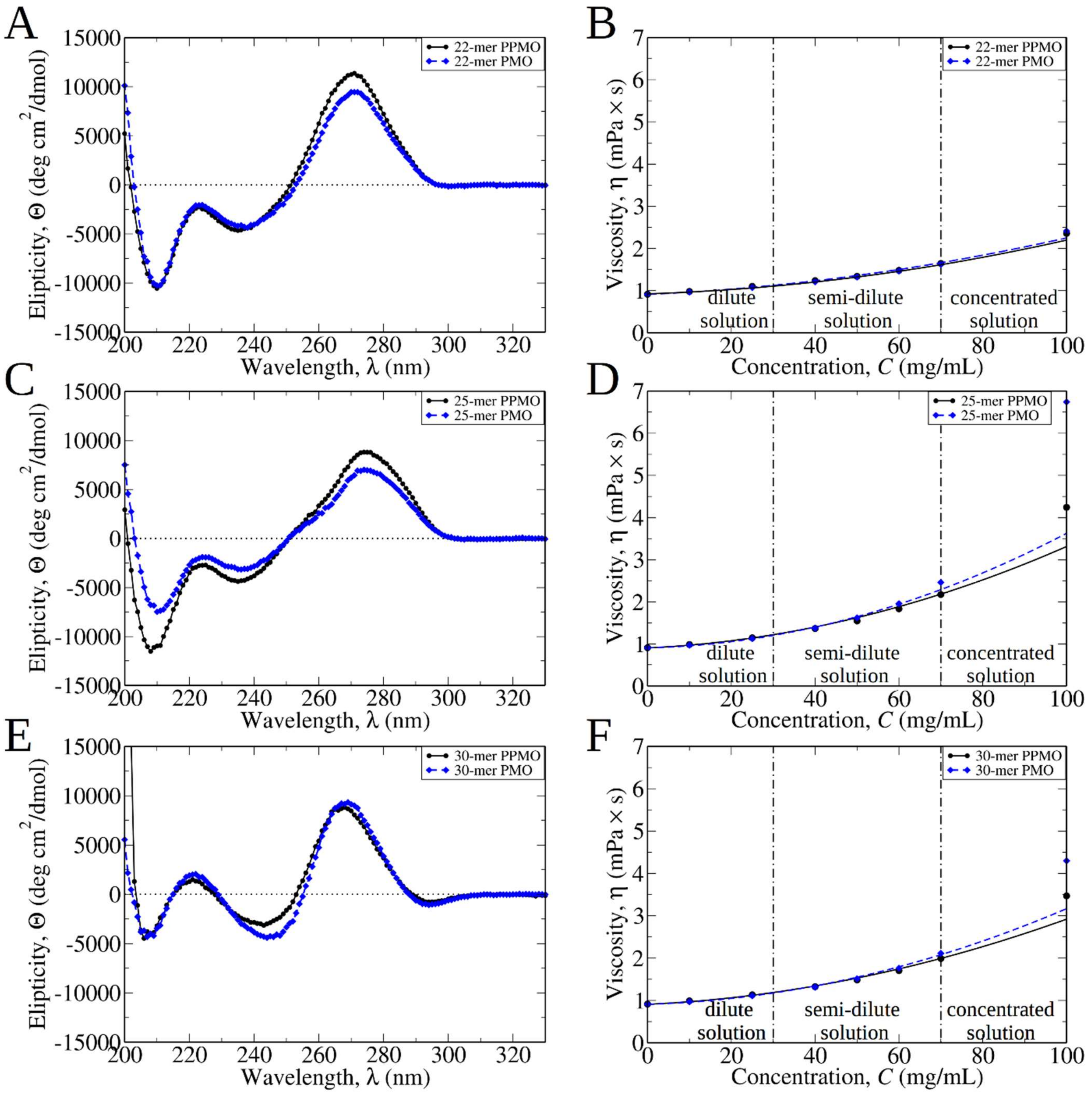

Figure S4. Comparison of CD spectra and viscosity profiles for 22-mer, 25-mer, and 30-mer PPMOs and PMOs: Superposed are the experimental CD profiles for the 22-mer PPMO and PMO (panel **A**), 25-mer PPMO and PMO (panel **C**), and 30-mer PPMO and PMO (panel **E**), and the 25°C viscosity η vs. concentration C profiles for the 22-mer PPMO and PMO (panel **B**), 25-mer PPMO and PMO (panel **D**), and 30-mer PPMO and PMO (panel **F**). For the PPMOs (PMOs), the black circles (blue diamonds) are connected with the black solid curve (dashed blue curve).

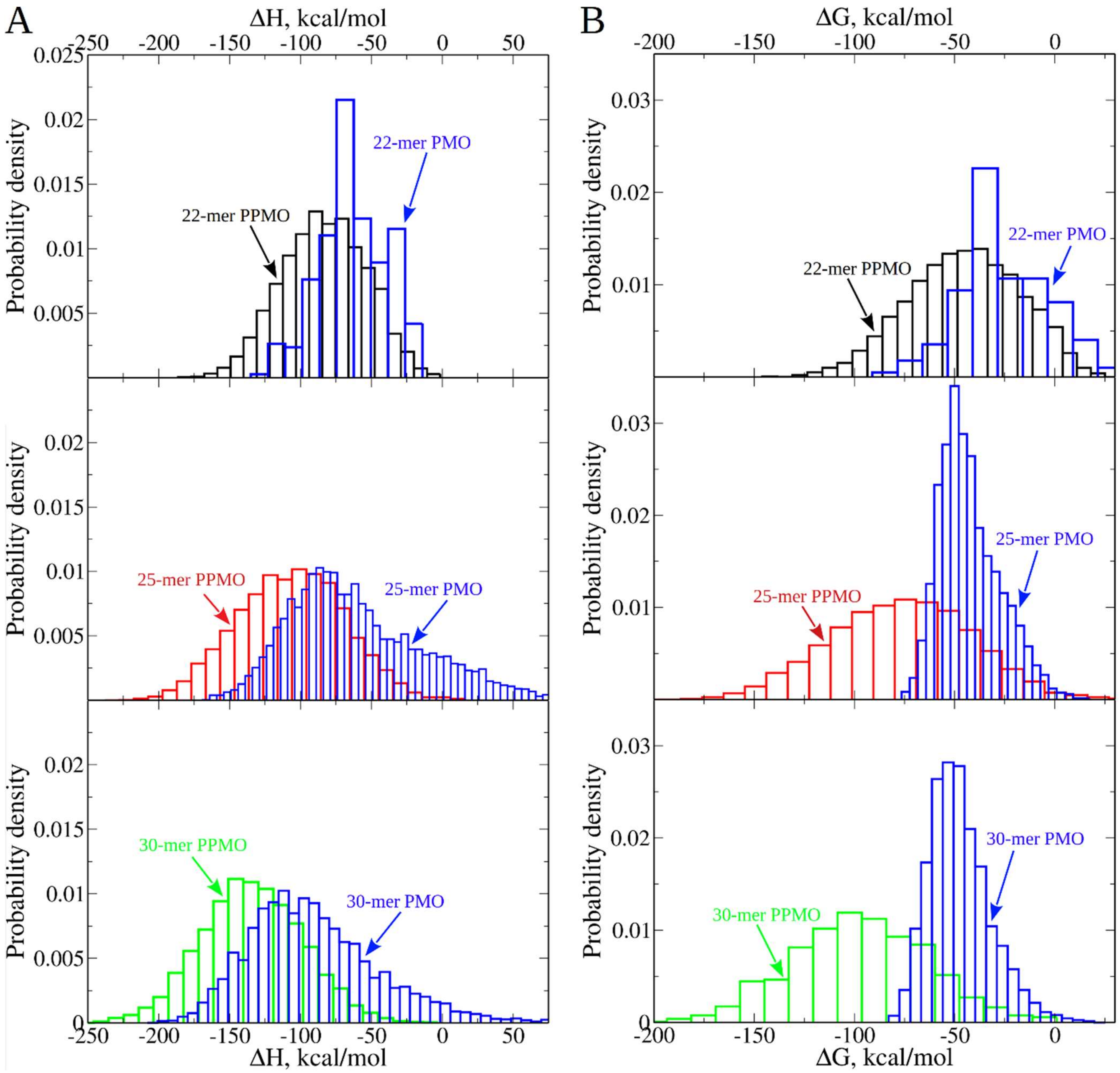

Figure S5. Histograms of thermodynamic state functions for 22-mer, 25-mer, and 30-mer PMOs and PPMOs: Shown are the histogram-based estimates of the normalized probability density functions (probability distributions) for the enthalpy change ΔH (panel **A**) and free energy change ΔG (panel **B**) for folding of the 22-mer, 25-mer, and 30-mer PPMO (black, red, and green bars, respectively) and for folding of the 22-mer, 25-mer, and 30-mer PMO (blue bars) [9]. The probability distributions for the PPMOs were sampled using the output from the equilibrium MD simulations at $T = 300$ K temperature (see Materials and Methods in the main text). The probability distributions for PMOs were taken from our previous study [1].

Supplemental Movies

Video S1. Folding of the 25-mer PPMO: The movie shows the conformational transition in the 25-mer PPMO from the extended state to the collapsed state (folded state) as observed in a 190-ns MD simulation run at $T = 300$ K. The MD run was carried out in explicit water (cyan transparent spheres). The PPMO molecule is shown in the Licorice representation (sticks) and in the Twister representation for the PMO backbone (blue line). Peptide portion of PPMO is shown in cyan color. The MTEG -linker is shown in orange, A and T bases are shown in green, and C and G bases are shown in red. The length of the movie is 26 s (the movie is played ~137,000,000 times slower than the computational experiment).

Video S2. Conformational dynamics of the 25-mer PMO: The movie shows conformational fluctuations of the 25-mer in the folded state as observed in a 3-μs MD simulation run at $T = 300$ K. The MD run was carried out in explicit water (cyan transparent spheres). The PPMO molecule is shown in the Licorice representation (sticks) and in the Twister representation for the PMO backbone (blue line). Peptide portion of PPMO is shown in cyan color. The MTEG -linker is shown in orange, A and T bases are shown in green, and C and G bases are shown in red. The length of the movie is 44 s (the movie is played ~15,000,000 times slower than the computational experiment).

Supplemental references:

1. Maksudov, F., Kliuchnikov, E., Pierson, D., Ujwal, M.L., Marx, K.A., Chanda, A., Barsegov, V. Therapeutic phosphorodiamidate morpholino oligonucleotides: Physical properties, solution structures, and folding thermodynamics. *Mol Ther Acids*. 2023;31:631-647.
2. Cieplak, P., Cornell, W.D., Bayly, C., Kollman, P.A. Application of the multimolecule and multiconformational RESP methodology to biopolymers: Charge derivation for DNA, RNA, and proteins. *J Comput Chem*. 1995;16(11):1357-1377.
3. Vanquelef, E., Simon, S., Marquant, G., Garcia, E., Klimerak, G., Delepine, J.C., Cieplak, P., Dupradeau, F.Y. RED Server: a web service for deriving RESP and ESP charges and building force field libraries for new molecules and molecular fragments. *Nucleic Acids Res*. 2011;39(suppl_2):W511-W517.
4. Wang, J., Wang, W., Kollman, P.A., Case, D.A. Automatic atom type and bond type perception in molecular mechanical calculations. *J Mol Graph Model*. 2006;25(2):247-260.
5. Wang, J., Wolf, R.M., Caldwell, J.W., Kollman, P.A., Case, D.A. Development and testing of a general amber force field. *J Comput Chem*. 2004;25(9):1157-1174.
6. Qiu, D., Shenkin, P.S., Hollinger, F.P., Still, W.C. The GB/SA continuum model for solvation. A fast analytical method for the calculation of approximate Born radii. *J Phys Chem A*. 1997;101(16):3005-3014.

7. Darden, T., York, D., Pedersen, L. Particle mesh Ewald: An N· log (N) method for Ewald sums in large systems. *J Chem Phys*. 1993;98(12):10089-10092.

8. Pedregosa, F., Varoquaux, G., Gramfort, A., Michel, V., Thirion, B., Grisel, O., Blondel, M., Prettenhofer, P., Weiss, R., Dubourg, V., et al. Scikit-learn: Machine learning in Python. *J Mach Learn Res*. 2011;12:2825-2830.

9. Bura, E., Zhmurov, A., Barsegov, V. Nonparametric density estimation and optimal bandwidth selection for protein unfolding and unbinding data. *J Chem Phys*. 2009;130(1):015102.
