# Supplementary material for: Molecular properties and intramolecular interactions of peptide-conjugated phosphorodiamidate morpholino oligonucleotides
Source: Mol Ther Nucleic Acids. 2025 Aug 14;36(3):102685. doi: 10.1016/j.omtn.2025.102685 (PMC12408397; doi:10.1016/j.omtn.2025.102685)
Supplement: Document S2. Article plus supplemental information [file mmc4.pdf]

Molecular Therapy
Nucleic Acids
Original Article

Molecular properties and intramolecular interactions of peptide-conjugated phosphorodiamidate morpholino oligonucleotides

Evgenii Kliuchnikov,[1,3,5] Farkhad Maksudov,[1,4,5] Daniel Pierson,[2] Kenneth A. Marx,[1] Arani Chanda,[2] and Valeri Barsegov[1]

[1]Department of Chemistry, University of Massachusetts, Lowell, MA 01854, USA; [2]Technical Operations, Sarepta Therapeutics, Cambridge, MA 02142, USA

We combined circular dichroism (CD) and viscosity measurements with molecular dynamics (MD) simulations and classification and regression approaches to machine learning to characterize solution structures of 22-mer, 25-mer, and 30-mer peptide- (-GlyArg6) conjugated phosphorodiamidate morpholino oligonucleotides (PPMOs). PPMO molecules form non-canonical folded structures with 1.4- to 1.5-nm radius of gyration, 4–6 base pairs and 5–11 base stacks, characterized by −49 to −71 kcal/mol free energy of folding. The 4.5–6.1 cm^3/g intrinsic viscosity and Huggins constant of 4.5–9.7 indicate PPMO-PPMO interactions at higher concentrations. The random-coil 3′-end conjugated -GlyArg_6 portion does not alter molecular properties of phosphorodiamidate morpholino oligonucleotide (PMO) components, which explains why CD spectra, viscosity-concentration profiles, and inhibitor activities of 22-mer, 25-mer, and 30-mer PPMOs and PMOs are similar but the peptide enhances the PPMO cellular uptake. PPMOs' viscosity is lower than PMOs' viscosity, due to PMO-peptide position-dependent interactions, especially in 25-mer PPMO, explaining differences in CD and high-concentration viscosity. These results reiterate the importance of the conformational ensemble view of non-canonical PPMO structures in solution, in agreement with our previous PMO study. The addition of -GlyArg_6 does not alter the structure and molecular properties of the PMO components of the PPMO structures but impacts the viscosity of the PPMO-based aqueous solution formulations.

INTRODUCTION

Antisense oligonucleotides (ASOs) have been shown to be promising candidates for various therapeutic applications.[1] There are currently more than a dozen Food and Drug Administration-approved oligonucleotide-based therapies on the market.[1] While these oligonucleotides and those that are in clinical development include various modifications in backbone, ribose, and base to improve their overall efficacy, their delivery to the desired/targeted location has been a long-standing challenge for this class of compounds.[2] Different cellular delivery pathways have been developed for oligonucleotides—this includes chemical/covalent modifications such as conjugation of PEGs, GalNac, and the use of lipid nanoparticles that have been approved for various indications.[3] Another delivery approach, cell-penetrating peptides (CPPs), have been shown to be a promising delivery mechanism for oligonucleotides[4,5] and other therapeutic candidates.[6,7] A review of ASO delivery studies with incorporated CPPs via two primary vectorization approaches, covalent conjugation and nano particles formulation-based strategies, has been published.[8]

Phosphorodiamidate morpholino oligonucleotides (PMOs) are a subclass of ASOs that have the canonical nucleic acid backbone replaced by morpholino rings connected by phosphorodiamidate linkages.[9–11] Four PMOs have been approved for the treatment of Duchenne muscular dystrophy (DMD).[12–14] Use of CPP conjugated phosphorodiamidate morpholino oligonucleotides (peptide-conjugated PMO or PPMO) have been shown to improve their effectiveness. The first report of a significant improvement of PPMO over its PMO counterpart for treatment of DMD appeared in 2003,[10] where the HIV-Tat peptide was used to enhance delivery of PMOs. In the following years, multiple examples of cellular uptake of PMOs conjugated with a broader range of CPP-like modifications, arginine-rich peptides[15] and peptide-aminohexanoic acid combinations,[16] were reported. Compared to unmodified PMO, it was noted that PPMO had significantly improved muscle uptake, restored dystrophin and generally improved muscle pathology.[17,18] Extensive work on peptide nucleic acids/PMO internalization peptides, commonly known as Pips, conjugated PMOs has shown that the

Received 12 April 2025; accepted 11 August 2025;
https://doi.org/10.1016/j.omtn.2025.102685.

[3]Present address: Department of Biological Engineering, Massachusetts Institute of Technology, Cambridge, MA 02139, USA
[4]Present address: Department of Chemistry, University of Texas, Austin, TX 78712, USA
[5]These authors contributed equally
Correspondence: Arani Chanda, Department of Biological Engineering, Massachusetts Institute of Technology, Cambridge, MA 02139, USA.
E-mail: achanda@sarepta.com
Correspondence: Valeri Barsegov, Department of Chemistry, University of Massachusetts, Lowell, MA 01854, USA.
E-mail: valeri_barsegov@uml.edu

© 2025 The Authors.
Published by Elsevier Inc. on behalf of The American Society of Gene and Cell Therapy.
This is an open access article under the CC BY license (http://creativecommons.org/licenses/by/4.0/).

hydrophobic core of the naturally occurring Pip sequence plays a critical role for PMO delivery.[19,20] Use of another naturally occurring sequence, the DG9, a cell-penetrating peptide derived from human polyhomeotic 1 homolog (Hph-1) transcription factor, class of CPP, to improve delivery of PMOs against DMD and spinal muscular atrophy (SMA) targets, has been reported.[21] More broadly, arginine-rich sequences have been shown to be effective[22] at cellular delivery of ASOs.

Use of ASOs, including PPMOs, that are in different phases of clinical development for treatment of DMD has been reviewed in the literature (see Table 1 in Wilton-Clark and Yokota[23]). Specifically, PPMO has been shown to be capable of both improving the melting temperature values in target mRNAs[24] and exon skipping activities in specific muscles leading to improved muscle function.[25,26] PPMOs have also been shown to significantly improve the pharmacokinetic profile compared with PMOs in terms of effective exon skipping in target muscles, enhance potency at lower doses, and extend dystrophin restoration observed in DMD disease models.[17,18,27] A list of PPMOs and their therapeutic effects in experimental models have been reported.[28] As one example, the pharmacokinetic/pharmacodynamic (PK/PD) model of a PPMO in the DMD disease model has been published.[29,30]

From a stability standpoint, it has been shown that PMO-D-CPPs (D-isomers of CPP) have enhanced proteolytic stability over their naturally occurring L-isomer counterparts.[31] There have been discussions in the literature about the potential efficacy loss of D-amino acid replacements in CPPs in the cases where CPPs depend on a chiral interaction or higher ordered structure to enter the membrane.[32,33] Previous studies have indicated that the efficient CPPs for PMO delivery lack secondary structure and can enter the cell through alternative mechanisms.[34,35] Studies focusing on delivery of D-peptides have shown similar abilities in delivering a PMO to the nucleus of cells compared with the native L-isomer.[36] PMOs have been conjugated with a wide range of D-isomer modified peptides and peptide mimics to improve muscle tissue delivery and efficacy: including dendrimeric octaguanidine,[37] cyclic peptides with 2′O-Methyl sugar modifications of phosphorothioate backbones,[38] lipopeptides,[39] and cationic amphiphilic peptides.[40] This conjugation-enhanced efficiency leading to decreased dosing frequency is potentially a significant benefit for PMO-based DMD therapies. Current PMO therapies require weekly[12–14] dosing to achieve and sustain clinical benefits. While current home-based infusion procedures have alleviated some of the burdens of treatment, weekly treatments still require a considerable commitment of time and effort that makes a reduction in dosing frequency a desirable attribute.[26]

Considering the potential benefits of PPMOs compared with PMOs, it is notable that there is no information about the atomic structure of PPMOs (or PMOs), either from X-ray crystallography or nuclear magnetic resonance (NMR) experiments. The interatomic distance information available from the small angle X-ray scattering (SAXS) measurements allow one to probe the average shape and size of PPMO molecules, but not their atomic arrangements. In addition, these methods do not provide information about the conformational ensemble view of the PPMO molecules. This lack of structural information about the PPMO molecules at the atomic level of detail makes it challenging to link the experimentally observed molecular properties with the structural transitions occurring in these complex peptide- and PMO-containing molecules in aqueous solution. In our recent study,[41] we devised an approach that helped provide a first-ever view of PMO solution structure as embodied in the 22 nucleobases (22-mer), 25 nucleobases (25-mer), and 30 nucleobases (30-mer) therapeutic PMOs. We developed the atomic force field for the atoms forming the morpholino ring and the phosphorodiamidate group in the PMO backbone. This development enabled us to perform microsecond-long all-atom molecular dynamics (MD) simulations of these 22-mer, 25-mer, and 30-mer PMOs. By correlating the experimental CD spectra and viscosity-concentration profiles with those calculated theoretically using the MD simulations, we were able to resolve the ensembles of PMO conformers that exist in an aqueous solution for these three therapeutic PMOs at room temperature. While CD spectroscopy is widely used to gather basic knowledge about the chiral behavior and level and type of secondary structure of biomolecules (nucleic acids and proteins), the viscosity characterization is necessary in practical applications, e.g., to understand the tertiary structure propensities, including solution phase interactions.

In this study, we employed a similar approach as before with PMOs, combining the experimental CD spectroscopy and viscosity measurements, as structural signature tools, with computational molecular modeling to explore the dynamic molecular properties of the 22-mer, 25-mer, and 30-mer PPMOs (PMOs conjugated with 3′-end linked -GlyArg$_6$: all L-isomers)—three oligomers' sequences that are complementary to exon 45, exon 53, and exon 51, respectively, of the dystrophin gene pre-mRNA transcript[42.] For these PPMO molecules, we characterized the various metrics of their secondary structure (e.g., base pairs and base stacks) and tertiary structure (e.g., radius of gyration, end-to-end distance, and solvent-accessible surface area), their hydrodynamic properties (e.g., intrinsic viscosity and Huggins constant), and their thermodynamic state functions (free energy and enthalpy) for PPMO folding in solution. The results obtained helped us to explain the CD spectral signatures and trends observed for concentration-dependent viscosity, which are directly relevant to the manufacturability and injectability aspects of PPMO use as therapeutics.[43,44] The structural insights gained in this study advance the current understanding of the structure-function relationship for PPMOs. The multiple points of agreement we have obtained between experiments and theory validate our computational modeling approach as a predictive guide to better understanding PPMOs' properties and how they impact PPMO-based therapeutic applicability.

RESULTS

22-mer, 25-mer, and 30-mer PPMOs

The sequences of nucleotides forming the therapeutic 22-mer, 25-mer, and 30-mer peptide-linked phosphorodiamidate morpholino

A

**Exon45**: 5’-CAA TGC CAT CCT GGA GTT CCT G-3’ - GRRRRRR	22-mer 4A, 7C, **5G**, 6T (23%G)
**Exon53**: 5’-GTT GCC TCC GGT TCT GAA GGT GTT C-3’ - GRRRRRR	25-mer 2A, 6C, **8G**, 9T (32%G)
**Exon51**: 5’-CTC CAA CAT CAA GGA AGA TGG CAT TTC TAG-3’ - GRRRRRR	30-mer 10A, 7C, **6G**, 7T (20%G)

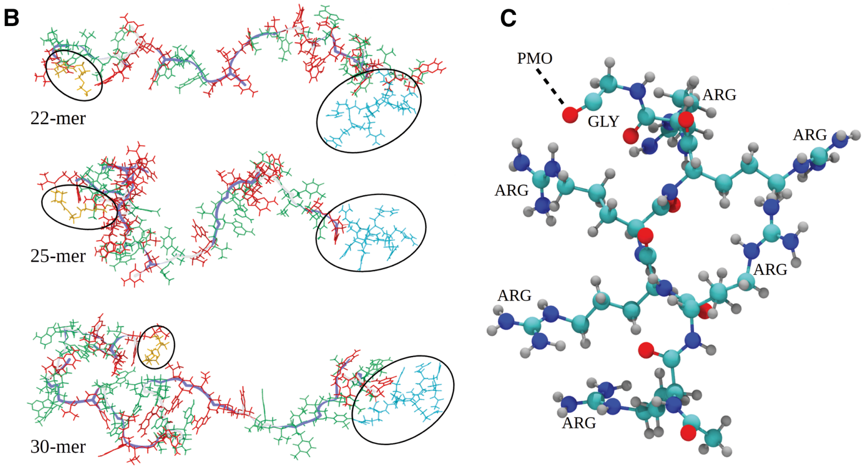

Figure 1. 22-mer, 25-mer, and 30-mer PPMO structures

Shown for three therapeutic PPMOs, exon 45 (22-mer PPMO), exon 53 (25-mer PPMO), and exon 51 (30-mer PPMO), are their PMO portion primary sequences, including the peptide part (A), and the reference structures – unfolded conformations (B). The PMO parts represent therapeutic oligomers complementary to the different indicated exon transcripts in the human dystrophin gene. Each sequence is shown with its base composition, the total amount of nucleobases, i.e., adenine (A), cytosine (C), guanine (G), and thymine (T), and the relative amount of G bolded (in percentage). The conformers are shown in Licorice representation (sticks) and in Twister representation (blue line) describing the backbone. The MTEG linker is shown in orange (within the black circle). The A and T bases are shown in green color, whereas C and G bases are shown in red. The seven-amino-acid peptide part of PPMO (-Gly-Arg$_6$) is shown in cyan (within the black circle). The more detailed view on the peptide part (C) shows that it is connected to the 3′-end of the PMO part at the C terminal of the glycine amino acid.

oligonucleotides (PPMOs), complementary to exons 45, 53, and 51, respectively, of the dystrophin gene pre-mRNA, are displayed in Figure 1A. Shown also are the total number of nucleobases (adenine, cytosine, guanine, and thymine) and the variable percent of guanine (G) in each sequence (varied between 20% and 32%). All PPMOs contain the morpholino triethylene glycol (MTEG) linker at the 5′-end of the molecule (see Figure 1B). It has been known that guanine-rich sequences form preferred secondary structures,[45] but since there are no more than two guanines next to each other in any of the PPMO structures, those secondary structures are not expected to form. The atomic structure of the 3′-end linked peptide is displayed in Figure 1C, which shows a pattern of six repeated Arg residues following a Gly residue (-GlyArg$_6$) residue.

Experimental CD spectra

First, we carried out CD spectroscopic measurements on the 22-mer, 25-mer, and 30-mer PPMOs (displayed in Figures 2A, 2C, and 2E) to explore their structures in aqueous solution. Because in our previous study of uncharged PMOs the CD spectra obtained for the molecules diluted in water and in DPBS were very similar,[41] in this study we describe only the CD spectra for the PPMO molecules in aqueous solution. The structures of PPMOs were studied by CD analysis in the UV wavelength region (200- to 330-nm range). The spectra of ellipticity Θ as a function of wavelength λ for all PPMO molecules are displayed in Figure 2. We see that the most significant band at $\lambda \approx 270$ nm and troughs at $\lambda \approx 210$ nm and 235–240 nm are observed for all PPMO samples. The intensities and exact peak and trough positions are only slightly different for individual PPMOs. The 270-nm peak amplitude decreases in the sequence: 22-mer > 25-mer > 30-mer PPMOs. By contrast, the 210-nm trough depth is maximal for the 25-mer, then decreases for the 22-mer, and is significantly lower for the 30-mer. The ∼270-nm peak, small 235- to 240-nm trough, and increasing positive values as the 200-nm measurement limit is reached is a characteristic feature of the chirality expressed by canonical right-handed helical RNAs.[46] As we showed in our prior study for PMOs,[41] addition of 1 M urea and 1 M LiCl to solutions of PMOs did not result in any changes to these peak maxima or their intensities (see Figure S4B in Maksudov et al.[41]). Because the helicity arrangements in PMO and PPMO molecules are expected to be very similar, these stable CD spectral features point to the overall stability and chiral similarity of the different members of the ensemble of PPMOs’ solution structures and their close similarity to the previously reported CD spectra of PMOs lacking CPP conjugation.[41]

Experimental viscosity-concentration profiles

Next, we profiled solution viscosity η as a function of mass concentration C for the 22-mer, 25-mer, and 30-mer PPMOs at 25°C temperature. At lower $C <$ 30 mg/mL concentrations (dilute solution regime), the viscosities of 22-mer, 25-mer, and 30-mer PPMOs were similar, equal to $\eta =$ 1.11 mPa/s, 1.15 mPa/s, and 1.13 mPa/s, respectively, for $C =$ 25 mg/mL concentrations (Figures 2B, 2D, and 2F). Interestingly, at higher 30–70 mg/mL concentrations (semi-dilute solution), the rate of change of η with C ($d\eta/_{dC}$), first increases for the 25-mer compared with the 22-mer PPMO, and then decreases for the 30-mer compared with the 25-mer PPMO. The solution viscosity of the 22-mer was lower overall compared with the 25-mer and 30-mer (Figures 2B, 2D, and 2F). Viscosity measurements for $C =$ 70 mg/mL PPMO solutions give $\eta =$ 1.64 mPa/s and 2.18 mPa/s for the 22-mer and 25-mer PPMOs, respectively, and $\eta =$ 2.00 mPa/s for the 30-mer PPMOs. Hence, the viscosity measurements not only revealed positive correlations of the solution viscosity with the concentration (and with the length of PMOs), but also the nonmonotonic dependence of $d\eta/_{dC}$ on the PMO sequence of

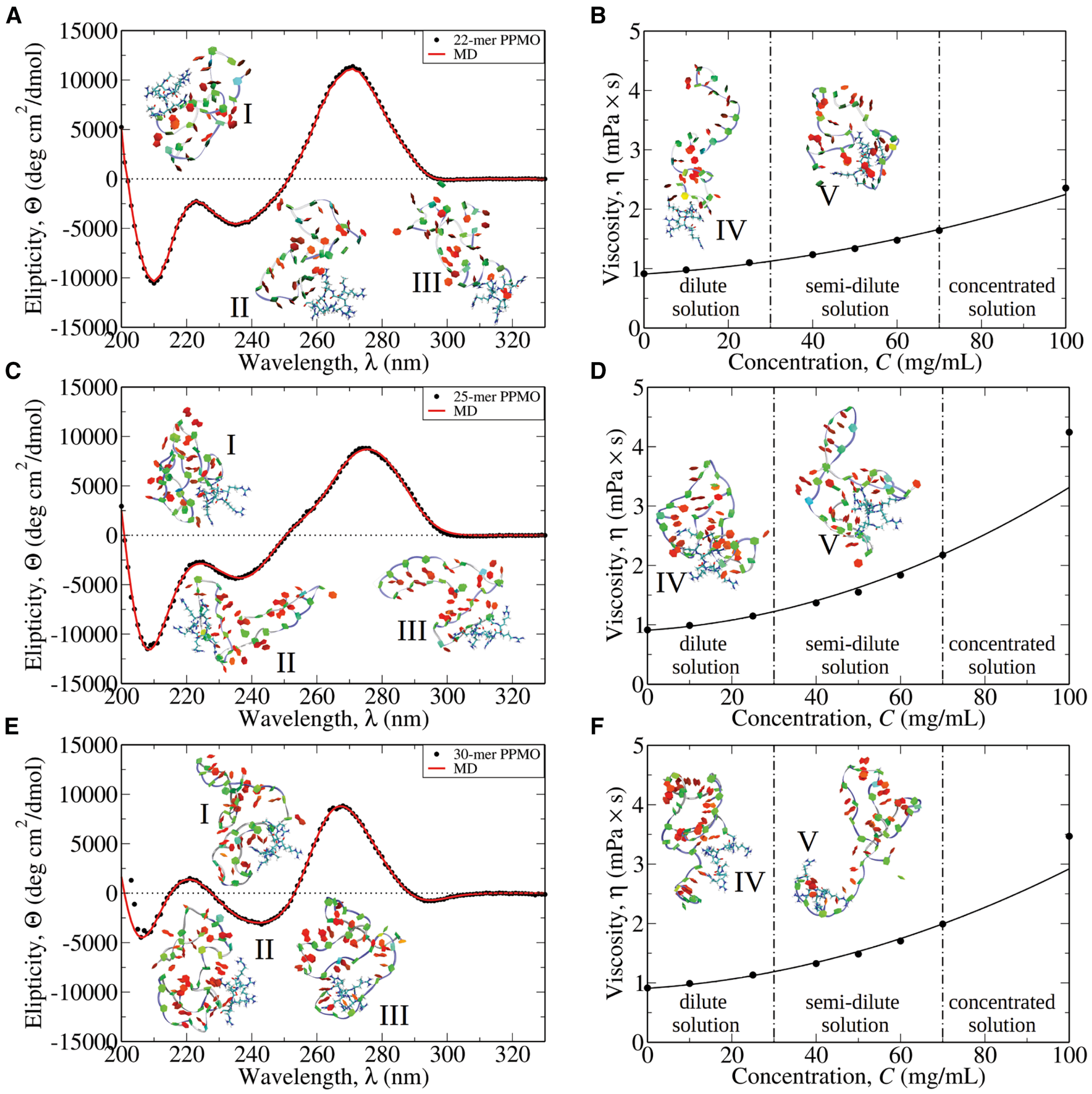

Figure 2. Theoretical reconstruction of CD spectra and viscosity profiles for 22-mer, 25-mer, and 30-mer PPMOs

Superposed are the CD profiles for the 22-mer (A), 25-mer (C), and 30-mer (E) PPMOs obtained experimentally (black dots) and theoretically (red solid lines), and the 25°C viscosity η vs. concentration C profiles for the 22-mer (B), 25-mer (D), and 30-mer (F) PPMOs obtained experimentally (black data points) and theoretically (black solid line). The snapshots of PPMO structures generated *in silico* numbered I–V, which correspond to the most representative, highest weight solution conformations contributing to the average CD spectra, are shown in Twister representation (blue line going through backbone) and in PaperChain representation (for nucleic bases).

PPMO molecules. This becomes more evident for PPMO concentrations above $C = 70$ mg/mL (concentrated solution); at higher $C = 100$ mg/mL concentration, $\eta = 2.36$ mPa/s and 4.24 mPa/s for the 22-mer and 25-mer PPMOs, respectively, and $\eta = 3.47$ mPa/s for the 30-mer PPMOs. Furthermore, in the concentrated solution regime ($C > 70$ mg/mL), the dependence of η on C becomes non-linear for all three PPMOs, which is indicative of the potential for dimerization, trimerization, and, possibly, formation of higher-order species at higher solution concentrations. Similar observations for the dependence of η on C have been reported, e.g., for PMO molecules[41] and monoclonal antibodies.[47]

Dynamic structural transitions in PPMO sequences

To provide a structural basis for interpretation of the experimental CD spectra and viscosity-concentration profiles (Figure 2), we turned to computational molecular modeling. In our prior work,[41] we have developed an atomic force field for PMOs, building upon the force fields available for nucleic acids (DNA and RNA).[48–51] In the present study, we have combined the force field for PMO with the force field for proteins to describe the molecular properties of the 22-mer, 25-mer, and 30-mer PPMOs (see supplemental information for more detail). For each PPMO molecule, we carried out 10 independent 1-μs long MD simulation runs

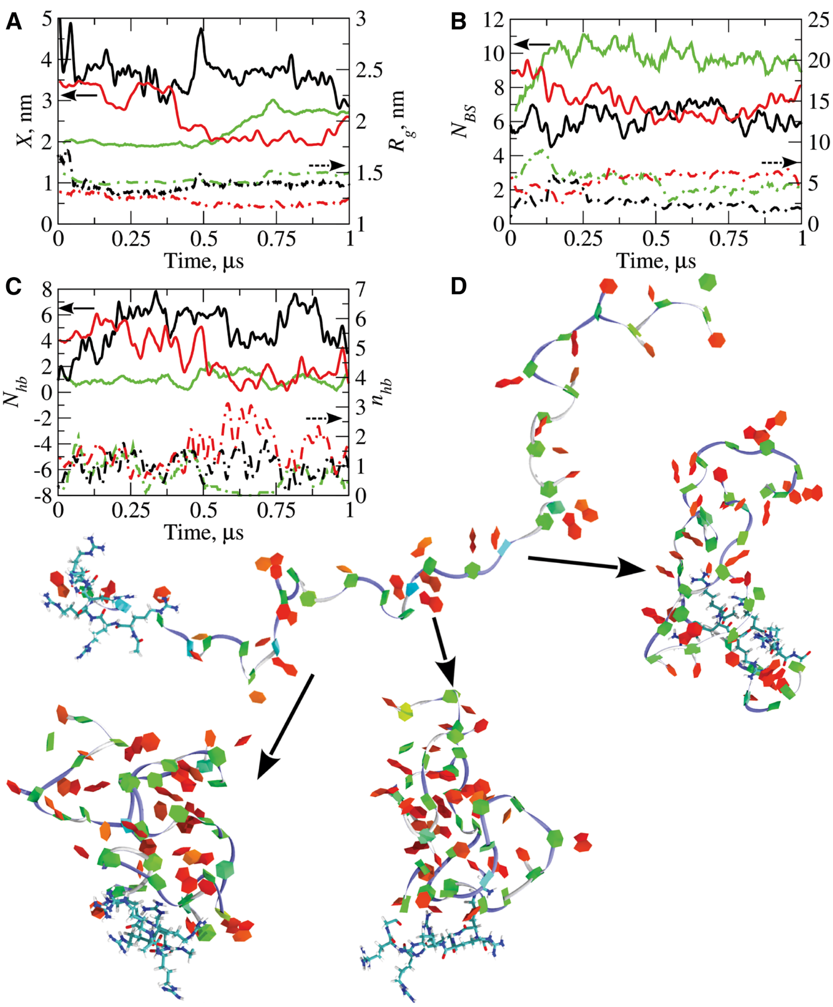

Figure 3. Dynamic structural properties and conformational transitions in 22-mer, 25-mer, and 30-mer PPMOs

(A–C) Time profiles of the structural properties of the 22-mer (black curves), 25-mer (red curves), and 30-mer (green curves) PPMOs from 1-µs MD simulation trajectories. (A) shows evolution of the end-to-end distance X (solid curves; left y-axis) and radius of gyration R_g (dashed curves; right y-axis). (B) shows the number of base-stacking interactions N_{BS} (solid curves; left y-axis) and base-pairing interactions N_{BP} (dashed curves; right y-axis). (C) displays the number of hydrogen bonds formed between the PMO and peptide parts of PPMO N_{hb} (solid curves; left y-axis) and number of hydrogen bonds formed within the peptide part only n_{hb} (dashed curves; right y-axis). (D) Extended conformation of the 30-mer PPMO and three representative partially folded conformations formed in the course of three independent MD runs. The extended and partially folded conformations are displayed in the Twister representation (for backbone; blue line) and in the PaperChain representation (for nucleic bases).

(materials and methods), starting from the extended conformations as initial structures (Figure 1). During the first 100–200 ns of the simulation, these extended conformations for all three PPMO molecules (see Figure 1B for several examples) transformed into more stable compact conformations of these molecules (Figure 3D). The reverse transition from the more compact structures to the more extended structures was not observed for any of the PPMOs studied. Folding transitions and conformational fluctuations for the 25-mer PPMO can be observed in Videos S1 and S2, respectively. Three representative examples of the partially folded conformations for the 30-mer PPMO are displayed in Figure 3D.

To better understand the structural changes associated with the folded conformations, we studied the following molecular properties of the 22-mer, 25-mer, and 30-mer PPMO molecules: the end-to-end distance X, the radius of gyration R_g, the number of base pairs N_{BP} and the number of base stacks N_{BS}. These important properties are readily accessible in the MD simulations (see supplemental information for more detail). While N_{BP} and N_{BS} provide information about the secondary structure of PPMO molecules, X and R_g contain information about the overall spatial distribution of atoms forming these molecules (tertiary structure). The time profiles of X and R_g, and N_{BP} and N_{BS} are depicted in Figures 3A and 3B, respectively, which show that these properties are variable. For example, the end-to-end distance fluctuates between X = 2.0 nm and 4.9 nm, and the radius of gyration fluctuates in the 1.3- to 1.5-nm range for the 22-mer, 25-mer, and 30-mer PPMOs (Figure 3A). These are small changes compared with the 16- to 22-nm length of extended PPMO molecules, implying limited variations in the tertiary structure. The number of base pairs N_{BP} varies between 1 and 7 and is smaller than the number of base stacks N_{BS}, which varies between 5 and 11 (Figure 3B). Nevertheless, these numbers are small compared with what is expected for a typical canonical folded single-stranded nucleic acid structure where a large fraction of bases are found in base-paired secondary structures.[52] Hence, the results obtained indicate that none of the PPMOs studied form significant portions of canonical nucleic acid duplex structure, which also can be gleaned from the structure snapshots (Figure 3D; see Video S1).

We also monitored the number of hydrogen bonds (H-bonds) formed between the hydrogen donor and hydrogen acceptor groups in the PMO and peptide portions N_{hb}, and within the peptide itself n_{hb}. While N_{hb} contains information about the interactions between the PMO and peptide components in the whole PPMO structure, n_{hb} reflects the propensity of the R6G peptide to form its own secondary structure. The profiles of N_{hb} and n_{hb} are displayed in Figure 3C, which shows N_{hb} = 1–8 H-bonds and n_{hb} = 1–3 H-bonds,

respectively. These results indicate that the -GlyArg$_6$ peptide interacts with the PMO structure but remains largely unstructured itself. This can also be gleaned from the structure snapshots (Figure 3D and Video S2).

Theoretical reconstruction of CD spectra

In our prior study, we used all-atom MD simulations to model the CD spectra for 22-mer, 25-mer, and 30-mer PMO molecules.[41] Here, we use the same approach to interpret the CD spectra for the 22-mer, 25-mer, and 30-mer PPMO molecules (see materials and methods). We used the structure output from MD simulations to select, for each 22-mer, 25-mer, and 30-mer PPMO molecule, the most dissimilar solution structures. We have gathered a total of 5,500 structures each for the 22-mer, for 25-mer, and for 30-mer PPMOs. These were used in the calculation of the theoretical CD curves. To resolve the most relevant solution structures of PPMOs and to evaluate their weights in the statistical ensemble of the structures selected, we performed a numerical fit of theoretical CD curves to the experimental CD profiles using non-linear regression (materials and methods). The procedure is described in detail in our prior study.[41] Briefly, for each i-th structure of a PPMO molecule, $i = 1, 2, \ldots, N$ ($N = 5{,}500$), we calculate a CD curve $\theta_i(\lambda)$. The average profile $\Theta_{th}(\lambda)$ is calculated as a superposition, $\Theta_{th}(\lambda) = \sum_i w_i \theta_i(\lambda)$, with w_i being the population weight for the i-th structure ($\sum_i w_i = 1$). Next, for each PPMO molecule, we use the mean squared error (MSE) as a penalty function and population weights $w_1, w_2, \ldots, w_N$ as regression coefficients to perform a numerical fit with the gradient descent algorithm (materials and methods). This enabled us to identify the primary (highest weights) solution structures for the 22-mer, 25-mer, and 30-mer PPMOs (and determine their weights) which best fit their experimental CD profiles. The average theoretical CD profiles $\Theta_{th}(\lambda)$ for the 22-mer, 25-mer, and 30-mer PPMOs are directly compared with the experimental CD spectra in Figures 2A, 2C, and 2E, respectively. The first five most important conformations I-V are shown as *the insets* in Figures 2A and 2B for the 22-mer PPMO, Figures 2C and 2D for the 25-mer PPMO, and Figures 2E and 2F for the 30-mer PPMO. These conformations account for 80%–90% of the equilibrium population of the 22-mer, 25-mer, and 30-mer PPMOs, respectively.

Properties of top three most important conformations of PPMO molecules

The excellent agreement between the theoretical CD curves and experimental CD spectra we have obtained enabled us to characterize the molecular properties of the first three most important, highest weight, conformations I, II, and III (Figures 2A, 2C, and 2E) for the 22-mer, 25-mer, and 30-mer PPMOs. For each conformation, the values of X and R_g, and N_{BP} and N_{BS} are shown in Table S1, which also lists the population weights. The end-to-end distance X varies between 1.7 nm and 3.2 nm for the 22-mer, between 1.3 nm and 2.3 nm for the 25-mer, and between 2.2 nm and 2.4 nm for the 30-mer PPMO. The radius of gyration R_g varies between 1.3 nm and 1.4 nm for the 22-mer, between 1.3 nm and 1.5 nm for the 25-mer, and between 1.4 nm and 1.5 nm for the 30-mer PPMO (Table S1). The number of base pairs N_{BP} varies between 2 and 9 for the 22-mer, between 2 and 5 for the 25-mer, and between 3 and 6 for the 30-mer PPMO. The number of base stacks N_{BS} varies between 4 and 5 for the 22-mer, between 7 and 10 for the 25-mer, and between 10 and 12 for the 30-mer PPMO (Table S1). We also calculated the solvent-accessible surface area $SASA$, which quantitates the degree to which a molecule is exposed to solvent (water). $SASA$ varies between 4,791 Å^2 and 5,314 Å^2 for the 22-mer, between 5,215 Å^2 and 5,763 Å^2 for the 25-mer, and between 6,179 Å^2 and 6,506 Å^2 for the 30-mer PPMO (Table S1).

Next, we calculated for conformers I–III the contributions to X, R_g, and $SASA$ from the separate PMO and peptide components of the total PPMO structure, which are compared in Table S1. For all three PPMOs, the PMO part of the PPMO structure provides larger contributions to the tertiary structure than the peptide part. For example, for conformers I–III, the values of R_g for the PMO are equal to the values of the same quantity for the full PPMO: for the 22-mer PPMO (1.3–1.4 nm) and for the 30-mer PPMO (1.4–1.5 nm) and are almost equal for the 25-mer PPMO (i.e., 1.3–1.4 nm for PMO vs. 1.3–1.5 nm for PPMO); see Table S1. The values of $SASA$ for the PMO are similar to the values for the full PPMO: for the 22-mer (3,613–3,967 Å^2 for PMO vs. 4,791–5,315 Å^2 for PPMO), for the 25-mer (4,632–4,911 Å^2 for PMO vs. 5,215–5,763 Å^2 for PPMO), and for the 30-mer (5,057–5,596 Å^2 for PMO vs. 6,179–6,506 Å^2 for PPMO); see Table S1. These results for R_g and $SASA$ indicate that the 3D structures of PPMO conformers are defined largely by the tertiary structures of their PMO components.

Theoretical reconstruction of viscosity-concentration profiles

Dilute and semi-dilute solutions

Next, we modeled the experimental solution viscosity data for the 22-mer, 25-mer, and 30-mer PPMOs. In the Einstein formula for the reduced solution viscosity, $\eta/\eta_s = 1 + [\eta]C + k_H[\eta]^2C^2$ (η_s is solvent viscosity; see materials and methods), the slope for the linear dependence term is given by the intrinsic viscosity $[\eta]$, which is the average inverse concentration of a molecule in its pervaded volume.[53] We used the 10 most populated conformations identified in the CD spectra analysis to calculate the values of intrinsic viscosity for the 22-mer, 25-mer, and 30-mer PPMOs. In Table S1, we list the values of η for the most important conformations I–III. These values vary within the same as well as different PPMOs, e.g., between 4.5 cm^3/g and 5.3 cm^3/g for the 22-mer, between 4.2 cm^3/g and 6.3 cm^3/g for the 25-mer, and between 4.3 cm^3/g and 6.1 cm^3/g for the 30-mer PPMO. Next, we used the results of MD simulations to calculate the theoretical average intrinsic viscosity, $[\eta]_{th} = \sum_i w_i \eta_i$, for the 22-mer, 25-mer, and 30-mer PPMOs (materials and methods). We selected the same top 10 conformations with the same weights extracted from theoretical analysis of the experimental CD spectra. The average values of $[\eta]_{th}$ came to 4.5 cm^3/g for the 22-mer, 4.6 cm^3/g for the 25-mer, and 6.1 cm^3/g for the 30-mer PPMOs (Table S2). We used these values of $[\eta]_{th}$ to predict the dependence of $\eta_{th}/\eta_s = 1 + [\eta]_{th}C$ on C in the dilute and semi-dilute solution regime for the 22-mer, 25-mer, and 30-mer PPMOs. We set

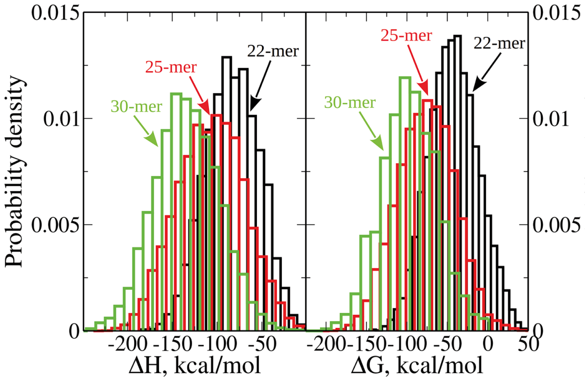

Figure 4. Thermodynamic state functions for 22-mer, 25-mer, and 30-mer PPMOs

Shown are the histogram-based estimates of the probability distributions of folding enthalpy ΔH (left) and folding free energy ΔG (right) for folding of the 22-mer PPMO (black bars), 25-mer PPMO (red bars), and 30-mer PPMO (green bars).[54] The distributions were sampled using the output from the equilibrium MD simulations at T = 300 K temperature and the unfolded solution structures as the reference states (displayed in Figure 1B). A total of 5,500 structures for each of the 22-mer, 25-mer, and 30-mer PPMOs were used in the thermodynamic analysis.

the water (solvent) viscosity to $\eta_s = 0.909$ mPa×s as estimated from the y-intercepts in the experimental viscosity profiles. Figures 2B, 2D, and 2F show excellent agreement between the experimental data and theoretical profiles of η for all PPMOs studied in the dilute and semi-dilute solution regimes. This validates our modeling approach and confirms our findings regarding the PPMO structures, behaving as noninteracting monomers, in the dilute and semi-dilute solutions.

Concentrated solution

Next, we described the viscosity data in the concentrated solution regime. By performing non-linear fits of the full Einstein formula, $\eta/\eta_s = 1 + [\eta]_{th}C + k_H[\eta]_{th}^2C^2$, to the experimental data with the values of $[\eta]_{th}$ estimated as described above, we obtained the Huggins constant k_H, which came to 4.5 for the 22-mer, 9.7 for the 25-mer, and 3.7 for the 30-mer PPMOs (Table S2). The theoretical curves of η vs. C deviate from the experimental data points in the concentrated solution regime for all PPMOs (Figures 2B, 2D, and 2F). In our previous study of PMOs, we found that at a higher concentration (> 75–100 mg/mL) the 22-mer, 25-mer, and 30-mer PMO molecules tend to begin interacting in an aqueous solution forming oligomers (dimers, trimers, etc.) These results point to interactions between PPMO molecules in concentrated solutions, which explains the differences between the theoretical results derived on the basis that PPMOs exist in the monomeric form and the upward deviating experimental data for the dependence of η on C for all three PPMOs (Figures 2B, 2D, and 2F).

Thermodynamic properties of PPMO molecules

Finally, we used the results of MD simulations to probe the thermodynamics of structural transitions in the 22-mer, 25-mer, and 30-mer PPMOs (materials and methods), and to resolve their folding enthalpy ΔH and folding free energy ΔG. For the most important folded conformations I–III, the values of ΔH and ΔG are accumulated in Table S1; in these calculations, we used the unfolded structures as reference states (see Figure 1B). For the complete ensembles of conformers, ΔH varies between −57 kcal/mol and −93 kcal/mol for the 22-mer, between −97 kcal/mol and −118 kcal/mol for the 25-mer, and between −104 kcal/mol and −114 kcal/mol for the 30-mer PPMOs. ΔG varies between −42 kcal/mol and −58 kcal/mol for the 22-mer, between −65 kcal/mol and −83 kcal/mol for the 25-mer, and between −60 kcal/mol and −84 kcal/mol for the 30-mer PPMOs (Table S1). In Table S1, we also list for all three PPMOs the contributions to the thermodynamic state functions from their PMO components. The values of ΔH and ΔG obtained show that the PMO portions contribute more significantly to the PPMO thermodynamic quantities compared with the peptide portions. The histogram-based estimates of the probability distributions of ΔH and ΔG for the 22-mer, 25-mer, and 30-mer PPMOs are displayed in Figure 4, which shows broad ΔH and ΔG distributions. Indeed, ΔH ranges from −186 kcal/mol to −1 kcal/mol for the 22-mer, from −238 kcal/mol to 16 kcal/mol for the 25-mer, and from −267 kcal/mol to −3 kcal/mol for the 30-mer PPMOs (Figure 4); ΔG varies between 49 kcal/mol and −146 kcal/mol for the 22-mer, between 59 kcal/mol and −208 kcal/mol for the 25-mer, and between 1 kcal/mol and −230 kcal/mol for the 30-mer PPMOs (Figure 4).

DISCUSSION

Therapeutic phosphorodiamidate morpholino oligonucleotides play an important role in the development of ASO-based approaches to drug discovery.[3] However, the relationship between their structure and function remains poorly understood, owing to the lack of any information about their solution structures. To date, there are no data from X-ray crystallography or NMR providing solution structure information of PMO molecules available to researchers. Yet, this information is critical for the development of rational drug design approaches to facilitate the creation of next-generation PMO-based applications. In our previous study,[41] we have overcome this lack of information about PMO solution structures by carrying out combined experimental and computational studies for the approved therapeutics, the 22-mer, 25-mer, and 30-mer PMOs. We showed that the all-atom MD simulations provide a powerful modeling tool to understand experimental results. Although computational molecular modeling has been used in the past to resolve molecular properties of RNAs and their derivatives,[55,56] our prior work was, to the best of our knowledge, the first systematic study of the solution structure and energetic molecular properties of therapeutic PMOs at the atomic level of detail.[41] Building on this initial success, in this study we took a step further, and we systematically explored composite peptide-linked phosphorodiamidate morpholino oligonucleotides (PPMOs), formed by the PMO molecules conjugated with a 3′-end linked -GlyArg$_6$ peptide (Figure 1).

Molecular properties of PPMOs

First, we carried out CD spectroscopic measurements on the 22-mer, 25-mer, and 30-mer PPMOs. CD spectroscopy has contributed to our understanding of the role of chirality in RNAs, due to the presence of favorable base-pairing and base-stacking interactions in the formation of A-form duplex RNA structures, as well as the contribution of helices, bulges, loops, and base mismatches to the overall tertiary structures of less structured RNAs.[57] All three PPMOs studied exhibited a rising positive CD signal as the lower experimental limit $\lambda \approx$ 200 nm was reached. This feature is characteristic of the right-handed chirality of helical RNA structures[46] and is similar to the behavior observed for the PMOs in our previous study.[41] All three PPMO molecules share similar spectral features above 200 nm, namely a large positive peak at $\lambda \approx$ 270 nm, a smaller magnitude trough at $\lambda \approx$ 240 nm, a small peak at $\lambda \approx$ 220 nm and a trough at $\lambda \approx$ 210 nm (Figures 2A, 2C, and 2E). All of these features are similar to an A-type RNA conformation with ordered right-handed base stacking.[52] The large positive peak intensities at $\lambda \approx$ 270 nm have the following order: 22-mer > 25-mer > 30-mer, while the small peak intensities at $\lambda \approx$ 220 nm have the opposite order: 22-mer < 25-mer < 30-mer. An important difference is that for the 22-mer and 25-mer PPMOs the ellipticity Θ is negative at $\lambda \approx$ 220 nm, while positive for the 30-mer PPMO (Figures 2A, 2C, and 2E). The negative peak at $\lambda \approx$ 210 nm present in all three PPMOs' spectra is likely due to the chirality properties of more localized electrons in stacked bases.[58] Any further interpretation of CD spectra of PPMO solutions requires analysis of their solution structures.

Second, we profiled the concentration dependence of viscosity of the PPMOs' solutions. The viscosity η dependence on PPMO concentration C is roughly linear in the 0- to 70-mg/mL range of dilute and semi-dilute solution concentration and is non-linear for concentrated solutions above 70 mg/mL (Figures 2B, 2D, and 2F). Structure analysis of the output from MD simulations shows that, because the PPMO molecules are uncharged, formation of partially ordered chiral secondary structure in monomeric PPMOs in the dilute and semi-dilute solutions is due to the interplay between the intramolecular hydrophobic and hydrophilic interactions relative to their interactions with solvent (Figure 3D), as we have discussed previously for PMOs.[41] As in the case of PMO molecules,[41] the origin of the higher concentration nonlinearity is potentially formation of higher-order structures between several PPMO molecules. Here too, the lack of any solution structure information about PPMO molecules made the interpretation of solution viscosity measurements difficult. This motivated us to perform the computational modeling of the PPMO molecules summarized below.

We carried out the all-atom MD simulations and employed the Support Vector Machines approach to machine learning to classify entire ensembles of conformations of the 22-mer, 25-mer, and 30-mer PPMOs in aqueous solution into either folded or unfolded structure classes. These conformations were used in conjunction with non-linear curve fitting (non-linear regression) to interpret the experimental CD spectra, in order to identify the top six to seven most important (most populated) solution conformations and to estimate their statistical weights (population percentages). These six to seven most important conformations account for ~90% of the ensemble of solution conformations for each PPMO. The top five conformations, I–V, for each PPMO, are displayed as the insets to Figure 2. Next, we explored the molecular parameters of the three most important conformers I–III of each PPMO, which are summarized in Table S1. These include N_{BP} and N_{BS} – metrics of the secondary structure content, $SASA$ – surface area of the molecule accessible to water, X and R_g – measures of overall tertiary structure, $[\eta]$ and k_H – measures of molecular viscosity and shape, and interaction propensity, respectively. For these same conformations I–III, we analyzed the thermodynamic properties enthalpy ΔH and free energy ΔG for folding (Table S1). Then, we used the six to seven most important PPMO conformations obtained from the modeling of the experimental CD spectra to calculate the average intrinsic viscosity $[\eta]$ for the 22-mer, 25-mer, and 30-mer PPMOs and to predict each PPMOs' solution viscosity η dependence on concentration C. This exercise can be viewed as an example of physics-based cross-validation used in statistical modeling of the PPMO solution structures. The excellent agreement between the experimental and theoretical curves for η vs. C (Figures 2B, 2D, and 2F) validates the computational modeling approach we have developed to describe the molecular properties of PPMOs. Moreover, using the six to seven top conformers, we obtained the statistics (average and standard deviations) of N_{BP}, N_{BS}, $SASA$, R_g, $[\eta]$ and k_H, and ΔH and ΔG for the 22-mer, 25-mer, and 30-mer PPMOs (Table S2).

Interestingly, the tertiary structure characteristic R_g does not change much (1.4–1.5 nm) with the number of bases (system size), while $SASA$ increases (from 5,247 Å^2 for the 22-mer to 6,555 Å^2 for the 30-mer PPMO) with the system size. This suggests that as the number of bases increases "the average properties of the solution structure ensemble" become more globular. The secondary structure metric N_{BP} decreases (from 6 for the 22-mer to 4.5 for the 30-mer PPMO), while N_{BS} increases (from 5.1 for the 22-mer to 10.9 for the 30-mer PPMO) with the number of bases (Table S2). These more sizable differences compared with that of R_g might result from differences in the PPMOs' sequence of bases. The intrinsic viscosity $[\eta]$, which quantitates the inverse concentration of a molecule in its pervaded volume, increases with the number of bases (from 4.5 cm^3/g for the 22-mer to 6.1 cm^3/g for the 30-mer PPMO), whereas the Huggins constant k_H, which can be indicative of a more extended shape of a molecule (as well as its being prone to intermolecular interactions in solution) first increases (from 4.5 for the 22-mer to 9.7 for the 25-mer PPMO) and then decreases (to 3.7 for the 30-mer PPMO); see Table S2. These last findings imply that, while the hydrodynamic properties of the PPMO molecules are expected to depend in some way on the number of bases, they also depend upon the different PMOs' nucleotide base compositions and/or their particular sequences. Elevated values of the Huggins constant are typically displayed by larger systems that are either globular but not flexible (e.g., BSA), that are rigid and extended in character (e.g., polystyrene sulfonate), or that are

aggregating (e.g., Folch-Pi protein, silica rods), to name a few properties and example systems.[59] Therefore, the large values of k_H we found for all PPMO molecules studied, especially for the 25-mer PPMO ($k_H = 9.7$), suggest that the ensemble of PPMO conformers we have identified possess a viscosity behavior that is driven by their shape and/or propensity for greater intermolecular interaction in solution.

Next, we calculated the average values of ΔH and ΔG using the same top six to seven most important 22-mer, 25-mer, and 30-mer PPMOs' conformers, which are accumulated in Table S2. These energy- and entropy-containing quantities characterize thermodynamic stability of the more compact partially folded structures as compared with their extended unfolded counterparts. Furthermore, using an ensemble of PPMO structures generated in the course of MD simulations (5,500 structures for each PPMO molecule), we mapped the entire distributions of these thermodynamic quantities (Figure 4). As expected from the thermodynamic state functions, ΔH and ΔG both vary with the system size, with ΔH decreasing (i.e., becomes larger and negative) from −86 kcal/mol for the 22-mer, to −103 kcal/mol for the 25-mer and 30-mer PPMOs (Table S2). This trend also can be observed in the histograms of ΔH, which shift toward larger negative values with increasing numbers of bases (Figure 4). The decrease in enthalpy ΔH correlates with the increase in the total number of base pairs and base stacks (i.e., $N_{BP} + N_{BS}$), from 11.1 for the 22-mer, to 13.3 for the 25-mer, and to 15.4 for the 30-mer PPMO, meaning that larger numbers of base pairs and especially base stacks correspond to stronger intramolecular interactions in these PPMOs. ΔG decreases from −49 kcal/mol for the 22-mer, to −71 kcal/mol for the 25-mer and to −70 kcal/mol for the 30-mer PPMOs (Table S2), implying the occurrence of more spontaneous folding transitions in these PPMOs. The histograms of ΔG also shift toward larger negative values with the increasing system size (Figure 4), implying that the thermodynamic stability increases in the sequence 22-mer PPMO < 25-mer PPMO ≈ 30-mer PPMO.

PMO-peptide and PMO-PMO interactions in PPMOs

We studied the separate contributions of PMO and peptide to the measured PPMOs' CD spectra. Using the 30-mer PPMO, we presented in Figure 5A the excellent agreement between the measured CD spectra and that calculated for the CD spectra from the five most representative structures shown. In Figure 5B, PMO and peptide contributions to the CD spectra were calculated from only the single structure shown (labeled 3, also in panel D). The peptide has no CD features above 240 nm and has a significant negative trough at ∼215 nm. Combining these two spectra in the ratio of 0.2 peptide to 0.8 PMO (their mass ratio in PPMO) produces a composite spectra that exactly matches the calculated PPMO spectra for this structure. This additivity behavior suggests that the peptide and PMO components produce minimal or no induced chiral CD spectral component in each other through potential interaction. This behavior may extend to many other solution structures, but perhaps not to all structures. To determine how the peptide component of PPMO behaves structurally, we present in Figure 5C the CD spectra for these five highest weight MD-simulated peptide structures (shown labeled in Figure 5D). They vary significantly in their chiral behavior, exhibiting both intensity and sign differences below 240 nm, but exhibit no clear secondary structure, such as the a-helix and b-sheet structures that have the respective CD spectra shown in Figure 5C (see *the inset* for the polyAla$_7$ and polyAla$_9$ with the a-helical structure, and for WW domain with the β-hairpin structure). These extended structures and the lack of secondary structure for the -GlyArg$_6$ peptide component of PPMOs are not simply based on their short length, but also are clearly dependent on their polycationic charge, since each Arg residue has a net + charge at pH 7 (pKa: 12.5[60]). We carried out determinations of the secondary structures present in the peptide during a total of 20 μs of MD (Figure S3), and the results show that the only predominant structures present are the Turn and Coil extended forms. The five structure examples taken from this result closely resemble the five labeled structures we examined in the CD spectral analysis (Figure 5D). Furthermore, the lack of any a-helix behavior in these simulations is likely given the short length and low ionic strength conditions used in these simulations.

Potential interactions between the PMO and peptide portions of PPMO molecules and their effect on the PMOs' potency are of interest for the design of new PPMO-based therapeutics. We present some representative examples of both clearly interacting and noninteracting PMO-peptide portions for structures observed in the MD simulations of 25-mer and 30-mer PPMOs (Figures 6A and 6B, respectively). For interacting PMO-peptide examples, a number of them show interactions at the 3′-end of the PMO, where covalent attachment occurs. While this might be expected due to their proximity, interactions also take place in some instances at PMO positions well away from the 3′-end, as a result of specific folding patterns occurring within the solution structures, bringing more distant PMO positions into close proximity to the 3′-end. To examine a more global, quantitative, and, therefore, comprehensive view of the PMO-peptide interactions in PPMOs, we analyzed the simulation outputs from ∼5,500 frames for each of the 22-mer, 25-mer, and 30-mer PPMOs.

We first examined the individual PMO base:Arg side chain interaction distribution along with PMO base:PMO base interactions in all three PPMOs (Figures 7A–7C). These 2D maps for each of the PPMOs show the PMO base:PMO base and PMO base:Arg side chain interaction pair time fraction % values. These plots represent the interactions of all types using a 7.5-Å interaction distance cutoff. In these plots (and in Figures 7D–7F), the residues are labeled starting at each PMOs' 3′-end, going from left to right on the *x*-axes, opposite to the PMOs' orientation shown in the Figure 1 representation, and the sequence of each PPMO molecule is shown below and aligned with the position numbered *x*-axes. Most of the longest interaction time fraction PMO base:PMO base interactions occur between nearest neighbors along the sequence (Figures 7A–7C). For example, in the 25-mer, these interactions are more numerous and

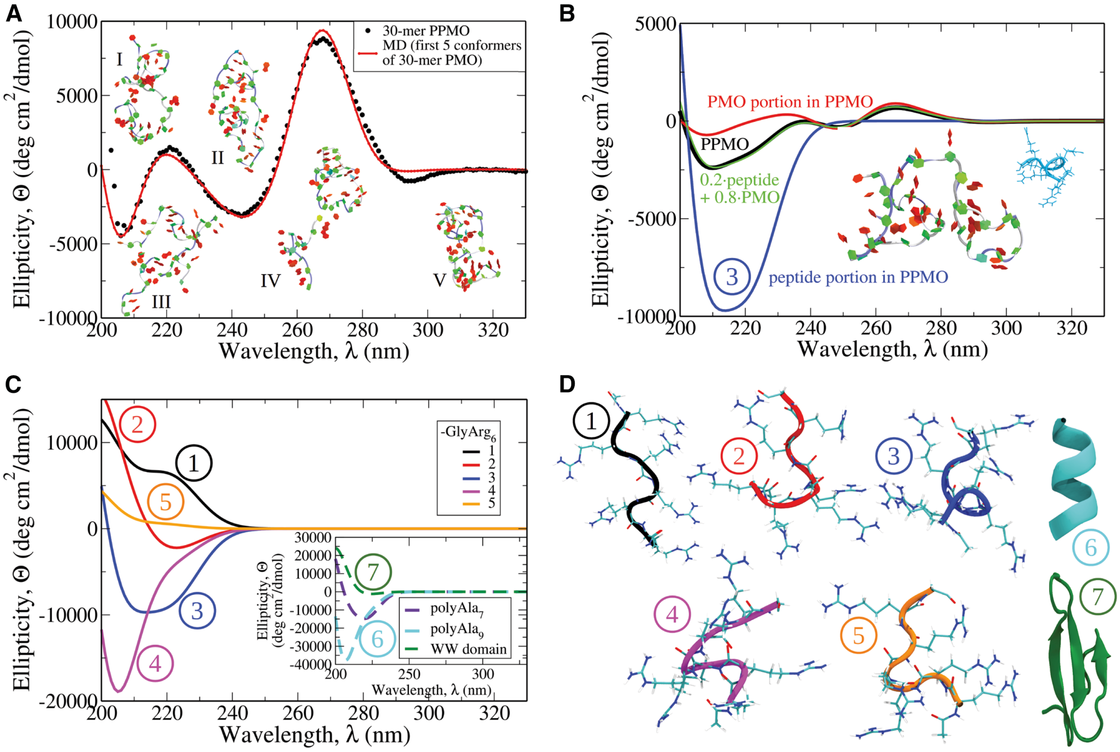

Figure 5. Role of peptide in CD spectral signals for PPMOs

(A) shows the CD profiles for the 30-mer PPMO (see Figure 1) obtained experimentally (black dots) and theoretically (red solid lines) using only structures from five of the most representative, highest weight solution PMO conformations obtained from our previous study.[41] The snapshots of PMO structures generated *in silico* numbered I–V, which correspond to contributing to the average CD spectra, are shown in Twister representation (blue line going through backbone) and in PaperChain representation (for nucleic bases). (B) displays the CD spectra of a 30-mer PPMO, which can be represented as a linear combination of PMO and peptide parts with the weights proportional to their sizes. Shown are the CD profile for a PPMO structure (black curve), for the peptide part (blue curve) and PMO part (red curve) of PPMO, and linear combination of PMO and PPMO parts (green curve). (C) displays the CD spectra for the peptide part of PPMO; solid lines of different colors represent several randomly selected structures of the peptide part of PPMO. The *inset* shows the CD profiles for some alpha-helical secondary structures: polyAla$_7$ (dashed purple line), polyAla$_9$ (dashed cyan line), and for the beta-sheet secondary structure of WW domain (dashed green line). (D) shows snapshots of the different peptides that were used for the CD spectra reconstruction in (B) and displayed in (C). The color code in the snapshots for all the structures in (D) corresponds to the colors of CD profiles in (C).

longest lived (pairs 1–2, 2–3, 5–6, 7–8, 8–9, 14–15, and 24–25) compared with the other two PPMOs. Also, for the 25-mer the next largest interaction difference compared with the other two PPMOs occurs at the off-diagonal position 15 interacting with positions 4–9. This interaction is relatively long lived and must result from one or more highly populated folds occurring in possibly many structures. One possible explanation for this clear 25-mer difference from the 22-mer and 30-mer PPMOs might lie in its base sequence difference. For the total PMO base:Arg side chain interactions (Figures 7A–7C), the 25-mer also stands out from the 22-mer and 30-mer in exhibiting the strongest interaction time fractions at the positions closest to the 3′-end. For instance, there is an especially long-lived interaction of PMO base at position 1 with Arg 2 and also Args 3–5. Similar position 2 peptide interactions with PMO position 1 exist in the 22-mer and 30-mers, but at shorter interaction time fractions. Out to PMO position 10 from the 3′-end, longer-lived interactions with specific peptide positions are evident in the 25-mer interaction map. The same 3′-end centered behavior of PMO base: Arg side chain interaction is evident for 22-mer out to PMO position 7 and 30-mer out to position 11, but these interactions are all shorter lived than for the 25-mer. These behaviors are all consistent with the evidence for long-lived folding interactions in the 25-mer PMO base: PMO base interaction time fraction panels compared with the 22-mer and 25-mer data we discussed above, and they point to the 25-mer as distinct from the 22-mer and 30-mer in its folding, exhibiting stronger and more persistent intramolecular interactions.

Next, we examined the quantitative interaction time fraction for all interaction types observed at each PMO base:Arg residue position, where the results are plotted vs. residue position (Figures 7D–7F). There is a maximum in interaction time fraction for PMO base: Arg side chain interactions for all three PPMOs starting at the 3′-end with position 1, then declining rapidly out to about position 10, where weaker more transient interactions occur at all remaining positions. Both the 25-mer and 30-mer have slightly higher levels of interaction time fraction compared with the 22-mer in this region.

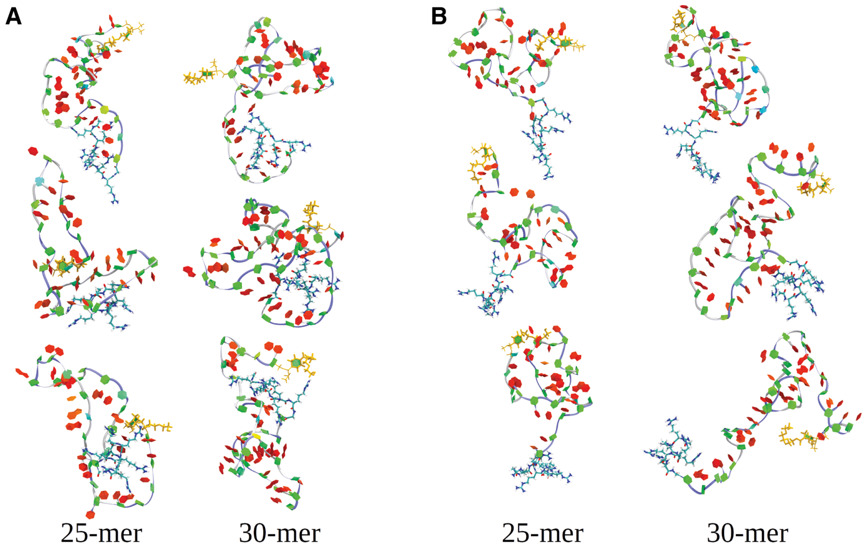

Figure 6. Diversity of conformations of 25-mer and 30-mer PPMO in aqueous solution

Displayed are structure snapshots for selected conformations of the 25-mer and 30-mer PPMO, in which the peptide parts interact with the PMO parts (A), and do not interact with the PMO parts (B). The snapshots are in the Twister representation (blue line going through backbone) and in PaperChain representation (for nucleic bases).

This general result is not surprising given the 3′-end attachment of the peptide, but the position-based details of the distribution of these interactions are different in the three PPMOs, as the data in Figure 7D indicate. Next, we examined a subset of all interactions, specifically the H-bond distributions for all three PPMOs. We determined the H-bonds made by peptide with bases and backbone portions of each PMO position separately (Figures 7E and 7F, respectively). For Arg H-bonds to both bases and backbone, the 25-mer has significantly (3- to 4-fold) higher total interaction time fractions (∼14%–30%) with the residues 1–5 close to the 3′-end compared with those for the 22-mer and 30-mer. For all three PPMOs, the interaction time fractions at all other positions vary between ∼7% and below, with the average being around 2%–3%. This low, but varying, interaction fraction at all positions results from the intramolecular interactions occurring within individual members of each PMOs' distribution of folded conformers.

In all three PPMOs, the largest intramolecular interaction time fractions for base:Arg side chain H-bonds are observed at positions where a pyrimidine base occurs. A preponderance, 9 of 10 such H-bonds, above 5% interaction time fraction occur at positions where a pyrimidine base is found, while 6 of 7 positions where such H-bonds are below 5% interaction time fraction occur where a purine base is found. For the 25-mer, all of the 5 H-bonds to either bases or backbone positions above ∼14% interaction time fraction occur at pyrimidine positions. This striking preference for base:Arg side chain H-bond formation and persistence at these pyrimidine positions in all three PPMOs suggest that a steric constraint may exist preventing ease of peptide interaction with purines due to the larger size of purines (two fused ring conjugated system) compared with pyrimidines (one ring conjugated system). Bolstering this interpretation is the fact that for most positions in all three PMOs where pyrimidines occur, the base:Arg side chain H-bond interaction time fraction > backbone-Arg side chain H-bond interaction time fraction. Yet, where purines occur, the reverse is true. This higher base:Arg side chain H-bond interaction profile at the 25-mer 3′-end compared with the 22-mer and 30-mer can be compared with the total interaction type profile from Figure 7D. Taking the numbers of base:Arg side chain H-bonds, these interactions represent 20% of the total interactions observed at positions 1 and 2 for the 25-mer, while the remaining 80% would be to varying degrees electrostatic in nature. However, for the 22-mer and 30-mer, the equivalent H-bond values for positions 1 and 2 together are only 2% and 3%, respectively, with the remainders of interactions being mainly electrostatic. Clearly, the 25-mer PPMO has a considerably different PMO:peptide interaction profile at its immediate 3′-end compared with 22-mer and 30-mer. Interestingly, the abundance of H-bonds at both base and backbone positions of the 25-mer would be expected, via competition, to lower the available sites for interaction with water solvent, thereby lowering the *SASA* values at these positions relative to the 22-mer and 30-mer.

PMO vs. PPMO

The addition of the 3′-end conjugated peptide to the PMO sequence to form PPMO structures alters the physical properties to some extent. Here, we describe the effect of peptide addition on the change in measured and calculated properties of these two structures, emphasizing the properties that could affect their manufacturability. First, as presented in Table S2 and described above, we have noted the difference in global tertiary structure properties of the 25-mer vs. the 22-mer and 30-mer for both the PMO and PPMO systems. The Huggins constant, k_H, was especially high for both PMO and PPMO 25-mers compared with the 22-mer and 30-mers (Table S2). One aspect of the Huggins constant interpretation is that higher values are associated with intermolecular interaction behavior. Since the intermolecular interactions giving rise to potential aggregation behavior are dependent upon the peptide addition and how it interacts with the PMO portion of the molecule, we directly compared both the measured CD and viscosity properties of the three PMOs vs. PPMOs.

Looking first at the CD comparisons (Figure S4), the 22-mer and 30-mer calculated and measured spectra for PMOs and PPMOs are in closest agreement, while the 25-mer shows small variation in

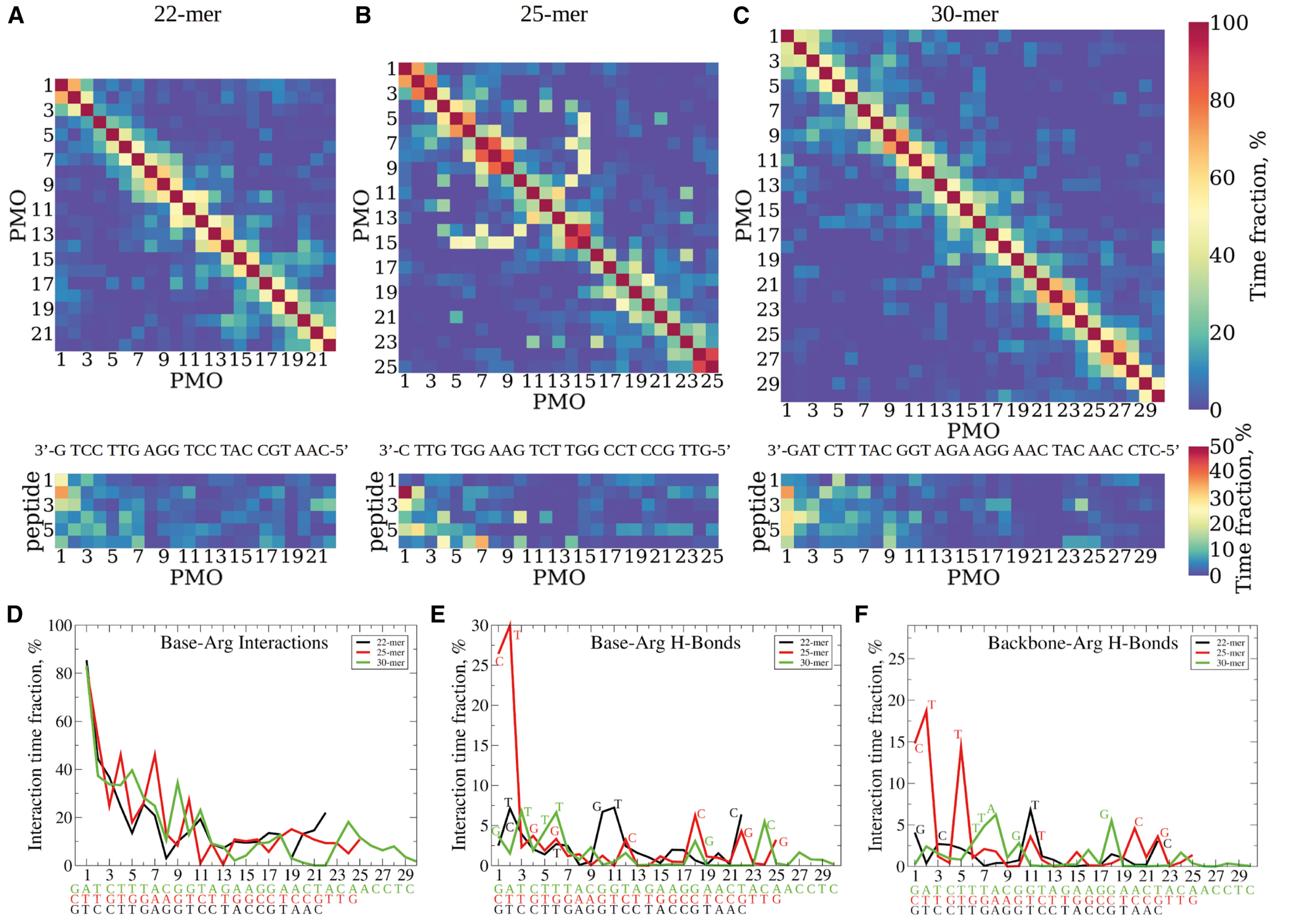

Figure 7. PMO-peptide interaction

(A–C) Intramolecular maps showing the time fraction of interactions between PMO bases (upper panels), and between PMO bases and peptide Arg side chains (lower panels) for the 22-mer (A), the 25-mer (B), and 30-mer (C) PPMOs. The numbering for both, PMO bases and six Arg amino acids starts from the 3′-end of the PMO portion. (D–F) provide the base-specific PMO-peptide interaction information showing the profiles of interaction time fraction as a function of the PMO base position for all the interactions between PMO bases and Arg side chains (A), the H-bonds formed between PMO bases and Arg amino acids (B), and the H-bonds formed between PMO backbone and Arg amino acids (C) in 22-mer (black), 25-mer (red), and 30-mer (green) PPMOs. The numbering for PMO bases starts from the 3′-end of the PMO sequence.

magnitude in all three spectral peak and trough regions. This 25-mer difference agrees with our overall discussion of the internal interaction behavior of Arg side chains with PMO bases and, especially, the 3′-end of the 25-mer PPMO having more interactions than that of the 22-mer and 30-mer. In PPMOs, the greater magnitude of the base:base, base:Arg side chain, and backbone:Arg side chain interactions for the 25-mer compared with 22-mer and 30-mer, suggest that the larger experimental CD differences (Figure S4) for 25-mer, especially in the negative 210-nm trough region, have their origin in these interaction differences; they produce alterations in chirality at varying wavelengths, for electrons in more localized (largely backbone) as well as delocalized (bases) bonding environments. When we next compare the viscosity profiles of PMOs vs. PPMOs (Figure S4), the behavior of both PMOs and PPMOs are very similar in the dilute and semi-dilute regions for all three molecules. However, in the concentrated region, where intermolecular interactions occur more frequently, the PMOs exhibit higher measured viscosity values compared with PPMOs in all cases. This relative behavior of peptide-containing vs. peptide-free PMOs is in general agreement with the use of Arg as a viscosity-lowering agent in drug formulations, although the mechanisms of action are different in these cases.[61] At the highest concentration measured, the viscosity difference is very small in the case of 22-mer, but becomes pronounced by the 25-mer, following the overall order: 25-mer > 30-mer > 22-mer. Again, these significant differences in PMO vs. PPMO behavior, especially for the 25-mer, can be explained by two things. First, their base:Arg side chain and base:base interactions, especially H-bonds, being substantially greater in the 25-mer vs. the 22-mer and 30-mer. Those greater 25-mer differences may produce molecular surface changes (peptide steric hindrance and water exposure) that are responsible for greater intermolecular interaction behavior in the 25-mer compared with the 22-mer and 30-mer, including in

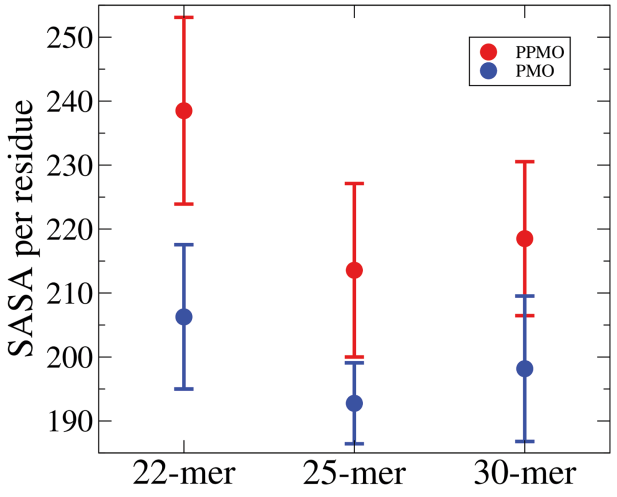

Figure 8. Solubility of 22-mer, 25-mer, and 30-mer PPMOs and PMOs
Shown is the scatterplot of the normalized value of *SASA* (averages and standard deviations) per nucleotide, representing solubility, for 22-mer, 25-mer, and 30-mer PPMOs (red; see Table S2) and for 22-mer, 25-mer, and 30-mer PMOs[41] (blue, see Table S2).

the case of PMO vs. PPMO. Second, PPMOs have the well hydrated charged peptide that the PMOs lack. These domains increase solubility for all PPMO species (see *SASA* values in Table S2; see also Figure 8) relative to their PMO counterparts, resulting in their lower-viscosity behavior.

Taking a different solubility-based view of the viscosity behavior of PMOs vs. PPMOs at high concentration, where aggregation can potentially occur, we next use calculated *SASA* values as determinants of interaction with solvent and, therefore, overall solubility. We determined the normalized *SASA* per residue for all three of the PMOs and PPMOs by dividing the total *SASA* (Table S2) by the number of nucleotide residues in each molecule. These normalized *SASA* per nucleotide values and standard deviations are plotted in Figure 8, which shows that for PMOs and PPMOs, both 25-mers and 30-mers have *SASA* per nucleotide residue values below that of their corresponding 22-mers. However, the 25-mers have the lowest values and therefore would be the least soluble in both the PMO and PPMO cases. This is in agreement with the 25-mer being potentially more prone to intermolecular interactions and, therefore, having the highest viscosity in both PMO and PPMO cases, which is consistent with it having the highest Huggins constant. Furthermore, the PMO *SASA* per residue value is significantly lower than that of the PPMO for the 25-mer, compared with the 22-mer and 30-mer differences. This is consistent with the highest viscosity difference being observed for the 25-mer (Figure S4D) at the highest concentration, with the PMO 25-mer exhibiting the highest viscosity of any molecule we studied; it also possesses a greater viscosity than the PPMO 25-mer at the highest concentration studied because of its lower *SASA* per nucleotide, therefore lower solubility and greater propensity for intermolecular interactions. The 30-mer exhibits behavior similar to that of the 25-mer, but the PMO and PPMO *SASA* per nucleotide values are not as low and not as different in magnitude as the 25-mer, resulting in viscosity behavior at the highest concentration studied for its PMO being moderately higher than for its PPMO (Figure S4F). The lowest *SASA* per nucleotide and, therefore, lowest solubility value observed for the 25-mer, relative to the 22-mer and 30-mer (Figure 8), is also consistent with its greater interactions with peptide discussed above and with its much higher H-bond density at the 3′-end, that would compete with and exclude water H-bonding possibilities.

From the energetics point of view, the incorporation of the 3′-end linked peptide to the PMO sequence results in stronger intramolecular PMO:PMO interactions (base pairs and base stacks) as well as PMO:peptide interactions (e.g., H-bonds), which in turn results, notwithstanding the associated entropy losses, in the increased stability against unfolding for all three 22-mer, 25-mer, and 30-mer PPMOs as compared with their PMO counterparts. This is reflected in the values of ΔH and ΔG for folding for the principal solution conformers I–III (Tables S1) as well as the average values of ΔH and ΔG (Table S2). This is also reflected in the histograms of the enthalpy changes ΔH (Figure S5A) and free energy changes ΔG for folding (Figure S5B) being shifted toward larger negative values of these quantities for all PPMO vs. PMO molecules (Figure S5). This explains the lower propensity of PPMO molecules for misfolding and intermolecular interactions in their concentrated aqueous solutions as compared with PMOs (Figure S4).

Conclusion

Three PPMOs, formed from 22-mer, 25-mer, and 30-mer PMO sequences, with the incorporation of a seven-amino-acid peptide, -GlyArg$_6$, at the 3-end of the PMO sequence, result in PPMO structures that are not much affected by the added peptide, possessing largely the structure and properties of their PMO components. The conformational ensemble perspective (a manifold of different solution structures, not a few selected structures) holds for PPMOs as determined by MD simulations, similar to what was observed previously in the case of PMOs.[41] Both for the PMO and peptide components, their conformational dynamics are defined by the competition between nonpolar groups for shielding from solvent exposure: in the case of the PMO, the backbone, comprised of nucleobases and uncharged phosphorodiamidate groups, and for the peptide, the peptide backbone. Similar structure-defining behavior was exhibited in the case of the nonpeptide containing PMOs. Although showing right-handed chirality by measured and calculated CD, none of the PPMOs studied form significant portions of canonical nucleic acid duplex structure.

The PPMO molecules form a manifold of stable, partially folded structures with a weak helical component, as well as extended structures exposing many of the bases externally to solvent in aqueous solution, though in many cases these exposed bases are stacked, thereby contributing to these structures' stability. This is behavior similar to that observed in the case of PMOs. The peptide portion of the

PPMOs is characterized by random-coil and turn structures under aqueous conditions of no added counterions. In the concentrated solution regime ($C > 70$ mg/mL), the dependence of η on C becomes non-linear for all three PPMOs, which is indicative of potential intermolecular interactions leading to dimerization, trimerization, and, possibly, higher-order interacting species. Again, this is similar to the viscosity behavior exhibited by PMOs. However, the viscosity of PPMO aqueous solutions is lower than the viscosity of their PMO counterparts, due to the lower solvent exposure of PMOs, as determined by MD simulations, producing a lower PMOs' solubility and greater propensity of PMOs to interact intermolecularly at higher concentrations. The importance of the morpholino oligonucleotides' base composition and specific sequences of the PMO portion of PPMOs (especially the 25-mer PPMO) resulted in the observation of somewhat different properties for the 25-mer compared with the 22- and 30-mers. These were as follows: slightly different CD intensities, higher viscosity and greater propensity to intermolecularly interact, and greater number of more persistent interactions between the bases and peptide Arg side chains, primarily at the 3′-end of the PMO portion. Overall, interesting sequence behavior was exhibited by the higher number of base:Arg side chain H-bond interactions with pyrimidine base positions compared with purines and the higher number of backbone:Arg side chain interactions at purine base positions at the 3′-end of all three PPMOs. Nevertheless, regardless of the morpholino oligonucleotides' base composition and sequence specific differences in these properties, all three 22-mer, 25-mer, and 30-mer PPMOs are uniformly characterized by stronger intramolecular interactions and are considerably more stable thermodynamically against unfolding compared with their PMO counterparts.

Considering that the -GlyArg$_6$ peptide addition to PMOs to generate PPMOs was carried out to facilitate cellular entry, and that PPMOs have been shown, for example the 30-mer PPMO, to be more effective against exon skipping compared with the corresponding sequence PMOs,[62] our own results show that the addition of -GlyArg$_6$ does not much alter the structure and molecular properties of the PMO components of the PPMO structures but impacts the viscosity of the PPMO-based aqueous solution formulations. Hence, it can be concluded that PPMOs are superior molecular entities for development; however, other factors, such as toxicity of peptide conjugates, should be considered in the context of therapeutic use.

MATERIALS AND METHODS

Synthesis of peptide-conjugated phosphorodiamidate morpholino oligonucleotides

Synthesis of peptide-conjugated phosphorodiamidate morpholino oligonucleotides (PMOs) comprising 22 nucleobases (22-mer), 25 nucleobases (25-mer), and 30 nucleobases (30-mer) (Figure 1) were performed using solid phase synthetic methodology described elsewhere.[9,10,15,16,63–65] All PPMOs were characterized including LC-MS for identity and high-performance liquid chromatography for overall purity. Other novel methods for the synthesis of PPMOs have been described in the literature.[66–69]

Experimental CD data

Circular dichroism (CD) measurements were carried out using a Chirascan Q100 Circular Dichroism Spectrometer with Pro-Data Viewer v.4.7.0.194 data analysis software (KBI Biopharma, Louisville, CO). The concentration of the sample was adjusted based on Beer's law to maintain an optimal absorbance signal of ∼0.8 AU. It was noted that the cell pathlength does not have a significant impact on the CD spectrum noise level, and hence a 1-cm pathlength cell was used for testing with a target concentration of 0.04–0.05 mg/mL, diluted in deionized water. The raw CD spectra were blank subtracted, baseline-corrected, and normalized to the mean residue molar ellipticity. Analysis was performed at 20°C temperature (Figure 2).

Experimental viscosity vs. concentration measurements

Experimental viscosity values were assessed for the 22-mer, 25-mer, and 30-mer PMOs. Concentrated stock solutions of each PMO were formulated by dissolving lyophilized drug substance material into distilled water. The concentration of the stock solutions was verified using UV-vis spectroscopy. A set of samples with concentrations ranging from 0 to 100 mg/mL PMO solutions were made from the stock solutions via serial dilution. The samples were analyzed for viscosity at 25°C temperature on a RheoSense VROC Initium Model #INI-H-1000. Analysis conditions were set to a controlled sample flow rate of 1,000 μL/min using the Initium high-pressure E02 chip. The Rheosense rheometric chip is thermally isolated and temperature controlled to ±0.1°C via micro heating elements and thermoelectric cooling systems. Prior to analysis, the Rheosense VROC Initium performs an automated system health check verifying hardware operational performance. Samples are controlled against a 10 cP glycerol standard manufactured by Paragon Scientific catalog number MGVS100-100ML. All reported sample results meet system suitability criteria for the weighted residual mean for the viscosity vs. shear rate curve of $R^2 \geq 0.999$. Samples were analyzed in quadruplicate with the average results reported. Estimated repeatability and accuracy error values are ±0.5% and ±2% respectively.

All-atom MD simulations

Atomic partial charges and force field parameters for PMO were derived in our previous work.[41] The all-atom MD simulations were carried out as described in the previous studies.[70,71] Briefly, each of the 22-mer, 25-mer, and 30-mer PPMOs was solvated in an octahedron water box, containing a PPMO wrapped with ∼10,300 water molecules (∼360 nm^3 volume) for 22-mer PPMO, ∼11,600 water molecules (400 nm^3 volume) for 25-mer PPMO, and ∼13,600 water molecules (∼470 nm^3 volume) for 30-mer PPMO, respectively. These simulation setups correspond to ∼50 mg/mL PPMO mass concentration. No counterions were included since PMOs are uncharged. We performed the energy minimization of each PPMO system, which involves two steps: first, using the steepest descent algorithm[72] (over 10,000 steps), and then using

the conjugate gradient method over 5,000 steps. In this step, the 50 kcal/mol energy restraint was applied to all solute atoms. Next, each PPMO system was heated from 0 K to 300 K within the 50-ps time. The equilibration for each PPMO was performed, in which 100 ps of restrained MD simulations were run with all solute atoms constrained with 0.05 kcal/mol energy. The final step included 1-μs-long unrestrained production MD simulation runs in water at T = 300 K for each PPMO study system using the CUDA version of pmemd[73] in the GPU-accelerated[72,74] AMBER 20 package.[75] To generate different dissimilar initial extended conformations for each of the 22-mer, 25-mer, and 30-mer PPMOs, with different orientations of bases along the backbone, but with the same end-to-end distance X, we ran 30-ns MD simulations with the first and last P-atoms constrained. The output data from MD simulations were analyzed as described in the supplemental methods.

Analysis of MD simulation output

The results of MD simulations for PPMOs (coordinate and energy files) were used in data analysis. *The end-to-end distance X* was calculated as the distance between the P-atoms of the first and last "morpholino nucleotides." *The radius of gyration* R_g was calculated using the coordinates of all atoms, $R_g = \left(\sum_p m_p \boldsymbol{r}_p^2 / \sum_p m_p\right)^{1/2}$, where m_p is the mass and $\boldsymbol{r}_p$ is the position of atom p, relative to the center of mass of the molecule. *The solvent-accessible surface area* (*SASA* was estimated using the LCPO algorithm[76] implemented in the CPPTRAJ module[77] in AmberTools20.[75] The total number of base pairs and number of base stackings were calculated using Barnaba software[42] with the structure schematic shown in Figure S1 guiding the calculation. *Base stacking:* If three conditions are satisfied: ($|z_{kj}|$ and $|z_{jk}| > 2$Å) and (ρ_{kj} or $\rho_{jk} < 2.5$Å) and ($|\theta_{kj}| < 40°$), bases were categorized as stacked. Here, $\rho_{kj} = \sqrt{x_{kj}^2 + y_{kj}^2}$, where the x- and y-axes lie in the plane of the base (x_{kj} and y_{kj} are the distances between the centers of mass of the two bases along the x- and y-axes, respectively) and the z-axis is perpendicular to the xy-plane, z_{kj} is the distance between the centers of mass of the two bases, and θ_{kj} is the angle between the normal vectors of the two bases (Figure S1).[42] *Base pairing:* All the non-stacked bases are considered to be base-paired if $|\theta_{kj}| < 60°$ and there is at least one hydrogen bond (H-bond) between k-th and j-th bases (Figure S1). *PMO-PMO and PMO-peptide interactions:* We assume that a pair of residues (nucleotides and amino acids) have interactions (electrostatic, hydrophobic, hydrogen bonds, etc.) if the distance between their centers of mass $d_{RES} < 7.5$Å-cutoff. The statistics of the distances was obtained via MDAnalysis Python package.[78,79] *Hydrogen bonds:* We assume that the H-bond D–H … A between the hydrogen donor atom (D) and acceptor atom (A) is formed if the donor–acceptor distance $d_{DA} < 3.3$Å-cutoff and if the bond angle is larger than the 140° cutoff.[42] To find the time fraction for each interaction, we used the H-bond option in CPPTRAJ module[77] implemented in AmberTools20.[75] *Structure similarity:* To prevent the selection of similar conformations of PPMOs, the eRMSD measure of structural similarity[80] implemented in the Barnaba software[42] was used. eRMSD is a contact map-based distance metric, with the addition of several features that make it suitable for the comparison of structures of nucleic acids. We used eRMSD to prescreen the output of the all-atom MD simulations for 22-mer, 25-mer, and 30-mer PPMO molecules, and to discard similar structures from subsequent data analysis.

Theoretical calculation of CD spectra for PPMOs

Theoretical calculation of a CD spectrum is implemented in the DichroCalc software.[81,82] For each 22-mer, 25-mer, and 30-mer PPMO conformer, we used the matrix method described in Johnson, Micsonai et al., and Chin et al.[83–85] to calculate theoretical CD spectra. This involves calculations of the interactions between different electronic excitations to determine the values of rotational strength. This results in a CD profile, which represents a set of the rotational strength values for each electronic transition as a function of the wavelength λ, $\theta(\lambda)$[82]. The software processes coordinate files as an input for CD spectra calculation. For PPMO, PMO, and peptide molecules, the CD spectra were determined using the output from molecular dynamics (MD) simulations (see Figures 5B and 5C).

Resolving solution conformations of PPMOs

We used the structure output from the all-atom MD simulations for the PPMO molecules in conjunction with non-linear curve fitting (non-linear regression) to the experimental CD spectra described in our previous study for PMO molecules.[41] In this study, we adapted this same approach[41] to interpret the experimental CD spectra for PPMO molecules. Here, we briefly describe the step-by-step implementation of the non-linear regression algorithm for theoretical reconstruction of the average theoretical CD spectrum $\Theta_{\rm th}$. The algorithm involves the following simple steps: *Step 1* is the assignment of a trainable weight (i.e., ensemble population) w_i to each CD profile θ_i representing the $i = 1, 2, \ldots, N$ conformation of a PPMO molecule in the ensemble. These weights are parameterized using a softmax transformation to ensure they remain non-negative and normalized ($\sum_i^N w_i = 1$). Initial values are sampled randomly from a uniform distribution. In *Step 2*, a weighted superposition is formed by combining the CD profiles with their pre-assigned weights ($w_1, w_2, \ldots, w_N$) from Step 1, i.e., $\Theta_{\rm th}(\lambda) = w_1\theta_1(\lambda) + w_2\theta_2(\lambda) + \ldots + w_N\theta_N(\lambda)$, in order to obtain the theoretical spectrum $\Theta_{th}(\lambda)$. In *Step 3*, the quality of fit is assessed by using the mean squared error (MSE) is calculated using the formula, $MSE = \frac{1}{m}\sum_{j=1}^{m}[\Theta_j(\lambda_j) - \Theta_{th\,j}(\lambda_j)]^2$, i.e., by comparing the values of experimental CD data point Θ_j and theoretical prediction $\Theta_{th\,j}$ for all wavelength values λ_j, $j = 1, 2, \ldots, m$ ($m = 131$ is the total number of data points covering the 200- to 330-nm range of a CD spectrum). In *Step 4*, the MSE is minimized by varying the populations w_i for all $i = 1, 2, \ldots, N$ conformations and by using the Adam optimizer[86] over 10,000 iterations. In *Step 5,* the overfitting is avoided by usage of two methods: (1) The softmax transformation, as a form of regularization, helps to constrain all conformational weights to be positive and normalized, thus preventing solutions when all weight is on a

single conformation unless **justified** by the data (loss function). (2) Because the fitting algorithm can produce multiple ensembles that match the experimental CD spectrum equally well (i.e., with similar *MSE*), we applied a thermodynamic criterion after fitting to select the most physically meaningful solution. Specifically, among all ensembles with comparable fits, we chose the one with the most negative weighted average folding free energy $\langle \Delta G \rangle = \sum_{i=1}^{n} w_i \Delta G / \sum_{i=1}^{n} w_i$. This step helps ensure that the final ensemble reflects the most thermodynamically stable set of conformations and prevents overinterpretation of solutions driven purely by numerical fit quality. In S*tep 6* the first $n = 5$–7 conformations with the largest weights (≥ 0.05), that account for ~85%–90% (majority) of all solution conformations of PPMOs, are selected for further analysis and modeling. This approach was used to model the CD spectra for the 22-mer, 25-mer, and 30-mer PPMOs, and to identify the five to seven PPMO conformations with the largest weights.

Theoretical reconstruction of viscosity-concentration profiles for PPMO solution

Reconstruction of the solution viscosity profile for each PPMO involves using the structure input, i.e., $n = 5$–7 largest-weight solution conformations identified in the theoretical reconstruction of CD spectra for PPMO molecules (see previous section). We used this same approach in our previous study for PMO molecules (see Maksudov et al.[41]). Briefly, for each i-th PPMO conformation, $i = 1, 2, \ldots, n$ (n is the total number of PPMO conformations), the intrinsic viscosity η_i values were calculated with the HYDROPRO package.[87] The theoretical average intrinsic viscosity was determined using the formula, $[\eta]_{th} = \sum_{i=1}^{n} w_i \eta_i / \sum_{i=1}^{n} w_i$, with ensemble populations (weights) w_i obtained from theoretical analysis of the experimental CD spectra (see previous section). Next, the profiles of PPMO solution viscosity η as a function of mass concentration C were obtained using the Einstein formula, $\eta = \eta_s(1 + [\eta]_{th} C + k_H [\eta]_{th}^2 C^2)$[88], where η_s is solvent viscosity and k_H is the Huggins coefficient. This approach was used to model the viscosity-concentration profiles for the 22-mer, 25-mer, and 30-mer PPMOs and to resolve the Huggins constant k_H for each PPMO.

Thermodynamic state functions

The free energy (ΔG) and enthalpy (ΔH) for folding of PPMO molecules were determined for each conformer observed in the equilibrium MD simulations for 22-mer, 25-mer, and 30-mer PPMO. The calculations were performed with the unfolded structures (Figure 1B) as the reference states. We used the molecular mechanics/generalized born surface area (MM/GBSA) method[89] implemented in the MMPBSA.py program[90] to analyze many thousands of conformations of the 22-mer, 25-mer, and 30-mer PPMOs. The enthalpy of a state H is estimated using the following contributions: $H = E_{int} + E_C + E_{vdW} + E_p + E_{np}$. In this equation, the first three terms are standard molecular mechanics potentials describing the bond length potential, bond angle potential, and dihedral angle potential (included in E_{int}), electrostatic interaction potential (E_C), and van der Waals interaction potential (E_{vdW}); E_p and E_{np} are the polar and nonpolar contributions to the solvation free energies, respectively. E_p is obtained by using the generalized Born (GB) model, and E_{np} is calculated using the solvent-accessible surface area *SASA*. We estimated the entropy of a state S using the normal mode analysis.[91] Changes in ΔH (enthalpy) and ΔS (entropy) were estimated by taking the difference between the enthalpies and entropies for a reference state (see Figure 1B) and the j-th conformation, i.e., $\Delta H_j = H_j - H_{ref}$ and $\Delta S_j = S_j - S_{ref}$, and ΔG_j was then calculated as $\Delta G_j = \Delta H_j - T\Delta S_j$ (for $T = 300$ K temperature). The procedure for creating the reference structures is described in the supplemental information.

DATA AVAILABILITY

All data are available from the corresponding authors upon reasonable request and included in the main text and supplemental information.

ACKNOWLEDGMENTS

We would like to thank Ms. Yuliia Gurova and Mr. Alexander Nikanshin for running the MD simulations for 22-mer PPMO, and Nikki Machalek (KBI Biopharma) for support with the circular dichroism data. This work was conducted under a Sponsored Research Agreement (SRA) between Sarepta Therapeutics and the University of Massachusetts, Lowell.

AUTHOR CONTRIBUTIONS

E.K.: Formal analysis, methodology, visualization, investigation, and writing − original draft. F.M.: Formal analysis, methodology, visualization, and investigation. D.P.: Formal analysis, investigation, visualization, and writing − original draft. K.A.M.: Conceptualization, formal analysis, investigation, methodology, supervision, validation, visualization, and writing − original draft. Arani Chanda: Conceptualization, formal analysis, investigation, methodology, supervision, validation, visualization, and writing − original draft. V.B.: Conceptualization, formal analysis, investigation, methodology, supervision, validation, visualization, and writing − original draft.

DECLARATION OF INTERESTS

D.P. and A.C. are employees of Sarepta Therapeutics Inc. and own stock/options in the company.

SUPPLEMENTAL INFORMATION

Supplemental information can be found online at https://doi.org/10.1016/j.omtn.2025.102685.

REFERENCES

1. Egli, M., and Manoharan, M. (2023). Chemistry, structure and function of approved oligonucleotide therapeutics. Nucleic Acids Res. *51*, 2529–2573.
2. Roberts, T.C., Langer, R., and Wood, M.J.A. (2020). Advances in oligonucleotide drug delivery. Nat. Rev. Drug Discov. *19*, 673–694.
3. Kulkarni, J.A., Witzigmann, D., Thomson, S.B., Chen, S., Leavitt, B.R., Cullis, P.R., and van der Meel, R. (2021). The current landscape of nucleic acid therapeutics. Nat. Nanotechnol. *16*, 630–643.
4. Derossi, D., Calvet, S., Trembleau, A., Brunissen, A., Chassaing, G., and Prochiantz, A. (1996). Cell internalization of the third helix of the Antennapedia homeodomain is receptor-independent. J. Biol. Chem. *271*, 18188–18193.
5. Hoyer, J.A.N., and Neundorf, I. (2012). Peptide vectors for the nonviral delivery of nucleic acids. Acc. Chem. Res. *45*, 1048–1056.
6. Khairkhah, N., Namvar, A., and Bolhassani, A. (2023). Application of cell penetrating peptides as a Promising drug carrier to combat viral infections. Mol. Biotechnol. *65*, 1387–1402.
7. Bottens, R.A., and Yamada, T. (2022). Cell-penetrating peptides (CPPs) as therapeutic and diagnostic agents for cancer. Cancers (Basel) *14*, 5546.
8. Lehto, T., Ezzat, K., Wood, M.J.A., and El Andaloussi, S. (2016). Peptides for nucleic acid delivery. Adv. Drug Deliv. Rev. *106*, 172–182.

9. Summerton, J., and Weller, D. (1997). Morpholino antisense oligomers: design, preparation, and properties. Antisense Nucleic Acid Drug Dev. *7*, 187–195.
10. Moulton, H.M., Hase, M.C., Smith, K.M., and Iversen, P.L. (2003). HIV Tat peptide enhances cellular delivery of antisense morpholino oligomers. Antisense Nucleic Acid Drug Dev. *13*, 31–43.
11. Xiong, Y., McQuistan, T.J., Stanek, J.W., Summerton, J.E., Mata, J.E., and Squier, T. C. (2018). Detection of unique Ebola virus oligonucleotides using fluorescently-labeled phosphorodiamidate morpholino oligonucleotide probe pairs. Anal. Biochem. *557*, 84–90.
12. FDA (2016). Drug Approval Package: Exondys 51 Injection (eteplirsen), p. 836.
13. FDA. Drug Approval Package: Vyondys 53 (golodirsen). Published online 2019.
14. FDA. Drug Approval Package: AMONDYS 45. Published online 2021.
15. Moulton, H.M., Nelson, M.H., Hatlevig, S.A., Reddy, M.T., and Iversen, P.L. (2004). Cellular uptake of antisense morpholino oligomers conjugated to arginine-rich peptides. Bioconjug. Chem. *15*, 290–299.
16. Abes, S., Moulton, H.M., Clair, P., Prevot, P., Youngblood, D.S., Wu, R.P., Iversen, P. L., and Lebleu, B. (2006). Vectorization of morpholino oligomers by the (R-Ahx-R) 4 peptide allows efficient splicing correction in the absence of endosomolytic agents. J. Control. Release *116*, 304–313.
17. Moulton, J.D., and Jiang, S. (2009). Gene knockdowns in adult animals: PPMOs and vivo-morpholinos. Molecules *14*, 1304–1323.
18. Moulton, H.M., and Moulton, J.D. (2010). Morpholinos and their peptide conjugates: therapeutic promise and challenge for Duchenne muscular dystrophy. Biochim. Biophys. Acta *1798*, 2296–2303.
19. Betts, C., Saleh, A.F., Arzumanov, A.A., Hammond, S.M., Godfrey, C., Coursindel, T., Gait, M.J., and Wood, M.J. (2012). Pip6-PMO, a new generation of peptide-oligonucleotide conjugates with improved cardiac exon skipping activity for DMD treatment. Mol Ther Acids *1*, e38.
20. Chwalenia, K., Oieni, J., Zemła, J., Lekka, M., Ahlskog, N., Coenen-Stass, A.M.L., McClorey, G., Wood, M.J.A., Lomonosova, Y., and Roberts, T.C. (2022). PPMO-mediated exon skipping induces uniform sarcolemmal dystrophin rescue with dose-dependent restoration of circulating microRNA biomarkers and muscle biophysical properties. Preprint at bioRxiv. https://doi.org/10.1101/2022.01.25.477672.
21. Haque, U.S., and Yokota, T. (2023). Enhancing Antisense Oligonucleotide-Based Therapeutic Delivery with DG9, a Versatile Cell-Penetrating Peptide. Cells *12*, 2395.
22. Turner, J.J., Ivanova, G.D., Verbeure, B., Williams, D., Arzumanov, A.A., Abes, S., Lebleu, B., and Gait, M.J. (2005). Cell-penetrating peptide conjugates of peptide nucleic acids (PNA) as inhibitors of HIV-1 Tat-dependent trans-activation in cells. Nucleic Acids Res. *33*, 6837–6849.
23. Wilton-Clark, H., and Yokota, T. (2023). Recent trends in antisense therapies for Duchenne muscular dystrophy. Pharmaceutics *15*, 778.
24. Nelson, M.H., Stein, D.A., Kroeker, A.D., Hatlevig, S.A., Iversen, P.L., and Moulton, H.M. (2005). Arginine-rich peptide conjugation to morpholino oligomers: effects on antisense activity and specificity. Bioconjug. Chem. *16*, 959–966.
25. Gushchina, L.V., Vetter, T.A., Frair, E.C., Bradley, A.J., Grounds, K.M., Lay, J.W., Huang, N., Suhaiba, A., Schnell, F.J., Hanson, G., et al. (2022). Systemic PPMO-mediated dystrophin expression in the Dup2 mouse model of Duchenne muscular dystrophy. Mol. Ther. Acids *30*, 479–492.
26. Gan, L., Wu, L.C.L., Wood, J.A., Yao, M., Treleaven, C.M., Estrella, N.L., Wentworth, B.M., Hanson, G.J., and Passini, M.A. (2022). A cell-penetrating peptide enhances delivery and efficacy of phosphorodiamidate morpholino oligomers in mdx mice. Mol. Ther. Acids *30*, 17–27.
27. Summerton, J. (1999). Morpholino antisense oligomers: the case for an RNase H-independent structural type. Biochim. Biophys. Acta *1489*, 141–158.
28. Tsoumpra, M.K., Fukumoto, S., Matsumoto, T., Takeda, S., Wood, M.J.A., and Aoki, Y. (2019). Peptide-conjugate antisense based splice-correction for Duchenne muscular dystrophy and other neuromuscular diseases. EBioMedicine *45*, 630–645.
29. Mukashyaka, M.C., Wu, C.L., Ha, K., Zhang, J., Wood, J., Foley, S., Mastis, B., Jungels, N., Sun, H., Shadid, M., et al. (2021). Pharmacokinetic/pharmacodynamic modeling of a cell-penetrating peptide phosphorodiamidate morpholino oligomer in mdx mice. Pharm. Res. *38*, 1731–1745.
30. Shadid, M., Badawi, M., and Abulrob, A. (2021). Antisense oligonucleotides: absorption, distribution, metabolism, and excretion. Expert Opin. Drug Metab. Toxicol. *17*, 1281–1292.
31. Youngblood, D.S., Hatlevig, S.A., Hassinger, J.N., Iversen, P.L., and Moulton, H.M. (2007). Stability of cell-penetrating peptide- morpholino oligomer conjugates in human serum and in cells. Bioconjug. Chem. *18*, 50–60.
32. Verdurmen, W.P.R., Bovee-Geurts, P.H., Wadhwani, P., Ulrich, A.S., Hällbrink, M., van Kuppevelt, T.H., and Brock, R. (2011). Preferential uptake of L-versus D-amino acid cell-penetrating peptides in a cell type-dependent manner. Chem. Biol. *18*, 1000–1010.
33. Knox, S.L., Wissner, R., Piszkiewicz, S., and Schepartz, A. (2021). Cytosolic Delivery of Argininosuccinate Synthetase Using a Cell-Permeant Miniature Protein. ACS Cent. Sci. *7*, 641–649.
34. Fadzen, C.M., Holden, R.L., Wolfe, J.M., Choo, Z.N., Schissel, C.K., Yao, M., Hanson, G.J., and Pentelute, B.L. (2019). Chimeras of cell-penetrating peptides demonstrate synergistic improvement in antisense efficacy. Biochemistry *58*, 3980–3989.
35. Schissel, C.K., Mohapatra, S., Wolfe, J.M., Fadzen, C.M., Bellovoda, K., Wu, C.L., Wood, J.A., Malmberg, A.B., Loas, A., Gómez-Bombarelli, R., and Pentelute, B.L. (2021). Deep learning to design nuclear-targeting abiotic miniproteins. Nat. Chem. *13*, 992–1000.
36. Schissel, C.K., Farquhar, C.E., Malmberg, A.B., Loas, A., and Pentelute, B.L. (2022). Cell-penetrating d-peptides retain antisense morpholino oligomer delivery activity. ACS Bio. Med. Chem. Au. *2*, 150–160.
37. Wu, B., Li, Y., Morcos, P.A., Doran, T.J., Lu, P., and Lu, Q.L. (2009). Octa-guanidine morpholino restores dystrophin expression in cardiac and skeletal muscles and ameliorates pathology in dystrophic mdx mice. Mol. Ther. *17*, 864–871.
38. Jirka, S.M.G., AC't Hoen, P., Diaz Parillas, V., Tanganyika-de Winter, C.L., Verheul, R.C., Aguilera, B., de Visser, P.C., and Aartsma-Rus, A.M. (2018). Cyclic peptides to improve delivery and exon skipping of antisense oligonucleotides in a mouse model for duchenne muscular dystrophy. Mol. Ther. *26*, 132–147.
39. Tajik-Ahmadabad, B., Polyzos, A., Separovic, F., and Shabanpoor, F. (2017). Amphiphilic lipopeptide significantly enhances uptake of charge-neutral splice switching morpholino oligonucleotide in spinal muscular atrophy patient-derived fibroblasts. Int. J. Pharm. *532*, 21–28.
40. Wang, M., Wu, B., Lu, P., Shah, S.N., Tucker, J.D., Bollinger, L.E., and Lu, Q. (2017). Evaluation of amphiphilic peptide modified antisense morpholino oligonucleotides in vitro and in dystrophic mdx mice. Polymers *9*, 177.
41. Maksudov, F., Kliuchnikov, E., Pierson, D., Ujwal, M.L., Marx, K.A., Chanda, A., and Barsegov, V. (2023). Therapeutic phosphorodiamidate morpholino oligonucleotides: Physical properties, solution structures, and folding thermodynamics. Mol. Ther. Acids *31*, 631–647.
42. Bottaro, S., Bussi, G., Pinamonti, G., Reißer, S., Boomsma, W., and Lindorff-Larsen, K. (2019). software for analysis of nucleic acid structures and trajectories. RNA *25*, 219–231.
43. Groves, M.J. (1989). Parenteral Technology Manual: An Introduction to Formulation, Production and Quality Aspects of Parenteral Products (Interpharm Press).
44. Overcashier, D.E., Chan, E.K., and Hsu, C.C. (2006). Technical considerations in the development of prefilled syringes for protein products. Am. Pharm. Rev. *9*, 77–83.
45. Sen, D., and Gilbert, W. (1988). Formation of parallel four-stranded complexes by guanine-rich motifs in DNA and its implications for meiosis. Nature *334*, 364–366.
46. Bloomfield, V.A., Crothers, D.M., and Tinoco, I. (2000). Nucleic Acids: Structures, Properties, and Functions (University Science Books).
47. Bravo-Anaya, L.M., Pignon, F., Martínez, F.A.S., and Rinaudo, M. (2016). Rheological properties of DNA molecules in solution: molecular weight and entanglement influences. Polymers *8*, 279.
48. Pérez, A., Marchán, I., Svozil, D., Sponer, J., Cheatham, T.E., III, Laughton, C.A., and Orozco, M. (2007). Refinement of the AMBER force field for nucleic acids: improving the description of α/γ conformers. Biophys. J. *92*, 3817–3829.
49. Zgarbová, M., Otyepka, M., Šponer, J., Mládek, A., Banáš, P., Cheatham, T.E., and Jurečka, P. (2011). Refinement of the Cornell et al. nucleic acids force field based

on reference quantum chemical calculations of glycosidic torsion profiles. J. Chem. Theory Comput. *7*, 2886–2902.

50. Zgarbová, M., Šponer, J., Otyepka, M., Cheatham, T.E., 3rd, Galindo-Murillo, R., and Jurečka, P. (2015). Refinement of the sugar-phosphate backbone torsion beta for AMBER force fields improves the description of Z-and B-DNA. J. Chem. Theory Comput. *11*, 5723–5736.

51. Cornell, W.D., Cieplak, P., Bayly, C.I., Gould, I.R., Merz, K.M., Ferguson, D.M., Spellmeyer, D.C., Fox, T., Caldwell, J.W., and Kollman, P.A. (1995). A second generation force field for the simulation of proteins, nucleic acids, and organic molecules. J. Am. Chem. Soc. *117*, 5179–5197.

52. Kloczewiak, M., Banks, J.M., Jin, L., and Brader, M.L. (2022). A Biopharmaceutical Perspective on Higher-Order Structure and Thermal Stability of mRNA Vaccines. Mol. Pharm. *19*, 2022–2031.

53. Doi, M., and Edwards, S.F. (1988). The Theory of Polymer Dynamics*73* (oxford university press).

54. Bura, E., Zhmurov, A., and Barsegov, V. (2009). Nonparametric density estimation and optimal bandwidth selection for protein unfolding and unbinding data. J. Chem. Phys. *130*, 015102.

55. Sponer, J., Bussi, G., Krepl, M., Banáš, P., Bottaro, S., Cunha, R.A., Gil-Ley, A., Pinamonti, G., Poblete, S., Jurečka, P., et al. (2018). RNA structural dynamics as captured by molecular simulations: a comprehensive overview. Chem. Rev. *118*, 4177–4338.

56. Golyshev, V.M., Abramova, T.V., Pyshnyi, D.V., and Lomzov, A.A. (2019). Structure and hybridization properties of glycine morpholine oligomers in complexes with DNA and RNA: Experimental and molecular dynamics studies. J. Phys. Chem. B *123*, 10571–10581.

57. Sosnick, T.R. (2001). Characterization of tertiary folding of RNA by circular dichroism and urea. Curr. Protoc. Nucleic Acid Chem. *Chapter 11*. Unit 11.5.

58. Gray, D.M., Liu, J.J., Ratliff, R.L., and Allen, F.S. (1981). Sequence dependence of the circular dichroism of synthetic double-stranded RNAs. Biopolymers *20*, 1337–1382.

59. Pamies, R., Hernández Cifre, J.G., del Carmen López Martínez, M., and García de la Torre, J. (2008). Determination of intrinsic viscosities of macromolecules and nanoparticles. Comparison of single-point and dilution procedures. Colloid Polym. Sci. *286*, 1223–1231.

60. Berg, J.M., Tymoczko, J.L., and Stryer, L. (2007). Biochemistry (Loose-Leaf) (Macmillan).

61. Ren, S. (2023). Effects of arginine in therapeutic protein formulations: a decade review and perspectives. Antib. Ther. *6*, 265–276.

62. Sarepta Therapeutics (2024). Sarepta Therapeutics Announces Positive Data from Part B of MOMENTUM, a Phase 2 Study of SRP-5051 in Patients with Duchenne Muscular Dystrophy Amenable to Skipping Exon 51. https://investorrelations.sarepta.com/news-releases/news-release-details/sarepta-therapeutics-announces-positive-data-part-b-momentum.

63. Summerton, J.E., and Weller, D. (1993). Uncharged morpolino-based polymers having phosphorous containing chiral intersubunit linkages. U.S. Patent No. 5,185,444.

64. Weler, D., and Hassinger, J. (2011). Oligonucleotide analogs having cationic intersubunit linkages. U.S. Patent No. 7,943,762.

65. Sinha, S., Kundu, J., and Ghosh, U. (2024). Synthesis of fmoc-protected morpholino monomers and their use in the synthesis of morpholino oligomer. U.S. Patent No. 12,012,427.

66. Aviñó, A., Grijalvo, S., Pérez-Rentero, S., Garibotti, A., Terrazas, M., and Eritja, R. (2011). Synthesis of Oligonucleotide–Peptide Conjugates for Biomedical and Technological Applications. Methods Mol. Biol. 223–238. Published online.

67. Maruyama, R., and Yokota, T. (2022). Muscular Dystrophy Therapeutics: Methods and Protocols, *2587* (Springer Nature).

68. Venkatesan, N., and Kim, B.H. (2006). Peptide conjugates of oligonucleotides: synthesis and applications. Chem. Rev. *106*, 3712–3761.

69. Tung, C.H., and Stein, S. (2000). Preparation and applications of peptide- oligonucleotide conjugates. Bioconjug. Chem. *11*, 605–618.

70. Harikrishna, S., and Pradeepkumar, P.I. (2017). Probing the binding interactions between chemically modified siRNAs and human argonaute 2 using microsecond molecular dynamics simulations. J. Chem. Inf. Model. *57*, 883–896.

71. Bottaro, S., Bussi, G., Kennedy, S.D., Turner, D.H., and Lindorff-Larsen, K. (2018). Conformational ensembles of RNA oligonucleotides from integrating NMR and molecular simulations. Sci. Adv. *4*, eaar8521.

72. Salomon-Ferrer, R., Götz, A.W., Poole, D., Le Grand, S., and Walker, R.C. (2013). Routine microsecond molecular dynamics simulations with AMBER on GPUs. 2. Explicit solvent particle mesh Ewald. J. Chem. Theory Comput. *9*, 3878–3888.

73. Darden, T., York, D., and Pedersen, L. (1993). Particle mesh Ewald: An N· log (N) method for Ewald sums in large systems. J. Chem. Phys. *98*, 10089–10092.

74. Le Grand, S., Götz, A.W., and Walker, R.C. (2013). SPFP: Speed without compromise—A mixed precision model for GPU accelerated molecular dynamics simulations. Comput. Phys. Commun. *184*, 374–380.

75. Case, D.A., Belfon, K., Ben-Shalom, I., Brozell, S.R., Cerutti, D., Cheatham, T., Cruzeiro, V.W.D., Darden, T., Duke, R.E., Giambasu, G., et al. Amber 2020 Reference Manual. 2020. Published online 2020.

76. Weiser, J., Shenkin, P.S., and Still, W.C. (1999). Approximate atomic surfaces from linear combinations of pairwise overlaps (LCPO). J. Comput. Chem. *20*, 217–230.

77. Roe, D.R., Cheatham, I.I.I., and PTRAJ, T.E. (2013). CPPTRAJ: software for processing and analysis of molecular dynamics trajectory data. J. Chem. Theory Comput. *9*, 3084–3095.

78. Michaud-Agrawal, N., Denning, E.J., Woolf, T.B., and Beckstein, O. (2011). MDAnalysis: a toolkit for the analysis of molecular dynamics simulations. J. Comput. Chem. *32*, 2319–2327.

79. Gowers, R.J., Linke, M., Barnoud, J., Reddy, T.J.E., Melo, M.N., Seyler, S.L., Domanski, J., Dotson, D.L., Buchoux, S., Kenney, I.M., et al. (2016). MDAnalysis: a Python package for the rapid analysis of molecular dynamics simulations. Proc. Python Sci. Conf. *98*, 105.

80. Bottaro, S., Di Palma, F., and Bussi, G. (2014). The role of nucleobase interactions in RNA structure and dynamics. Nucleic Acids Res. *42*, 13306–13314.

81. Bulheller, B.M., and Hirst, J.D. (2009). DichroCalc—circular and linear dichroism online. Bioinformatics *25*, 539–540.

82. Bulheller, B.M., Rodger, A., and Hirst, J.D. (2007). Circular and linear dichroism of proteins. Phys. Chem. Chem. Phys. *9*, 2020–2035.

83. Johnson, W.C. (1996). Determination of the Conformation of Nucleic Acids by Electronic CD. In Circular Dichroism and the Conformational Analysis of Biomolecules, G.D. Fasman, ed. (Springer Science & Business Media), pp. 433–468.

84. Micsonai, A., Wien, F., Kernya, L., Lee, Y.H., Goto, Y., Réfrégiers, M., and Kardos, J. (2015). Accurate secondary structure prediction and fold recognition for circular dichroism spectroscopy. Proc. Natl. Acad. Sci. USA *112*, E3095-–E3103.

85. Chin, S.L., Lu, Q., Dane, E.L., Dominguez, L., McKnight, C.J., Straub, J.E., and Grinstaff, M.W. (2016). Combined molecular dynamics simulations and experimental studies of the structure and dynamics of poly-amido-saccharides. J. Am. Chem. Soc. *138*, 6532–6540.

86. Kingma, D.P., and Ba, J. (2014). Adam: A method for stochastic optimization. Preprint at arXiv. https://doi.org/10.48550/arXiv.1412.6980.

87. Ortega, A., Amorós, D., and García de la Torre, J. (2011). Prediction of hydrodynamic and other solution properties of rigid proteins from atomic-and residue-level models. Biophys. J. *101*, 892–898.

88. Zuev, Y.F., Litvinov, R.I., Sitnitsky, A.E., Idiyatullin, B.Z., Bakirova, D.R., Galanakis, D.K., Zhmurov, A., Barsegov, V., and Weisel, J.W. (2017). Conformational flexibility and self-association of fibrinogen in concentrated solutions. J. Phys. Chem. B *121*, 7833–7843.

89. Hou, T., Wang, J., Li, Y., and Wang, W. (2011). Assessing the performance of the MM/PBSA and MM/GBSA methods. 1. The accuracy of binding free energy calculations based on molecular dynamics simulations. J. Chem. Inf. Model. *51*, 69–82.

90. Miller, B.R., 3rd, McGee, T.D., Jr., Swails, J.M., Homeyer, N., Gohlke, H., and Roitberg, A.E. (2012). MMPBSA. py: an efficient program for end-state free energy calculations. J. Chem. Theory Comput. *8*, 3314–3321.

91. McQuarrie, D.A. (2000). Statistical Mechanics (Sterling Publishing Company).

OMTN, Volume *36*

Supplemental information

Molecular properties and intramolecular interactions of peptide-conjugated phosphorodiamidate morpholino oligonucleotides

Evgenii Kliuchnikov, Farkhad Maksudov, Daniel Pierson, Kenneth A. Marx, Arani Chanda, and Valeri Barsegov

Supplemental Methods

Force field development for all-atom simulations of PPMO molecules: In our previous study [1], we derived the atomic partial charges and force-field parameters for the following three parts of the PMO structure: i) for the phosphorodiamidate group attached to the 5′-end and capping the HN atomic group attached to the N3′ atom (mimicking the PMO 5′-end); ii) for the phosphorodiamidate group attached to the 3′-end and capping the HO atomic group attached to the O5′ atom (mimicking the PMO 3′-end); and iii) for the phosphorodiamidate group attached to both 5′- and 3′-ends. In the same study [1], we also derived the force field parameters and partial charges for the morpholino triethylene glycol (MTEG) linker attached to the 5′-end of PPMO through the phosphorodiamidate group. For the peptide portion of PPMO, we used the original Amber ff14SB force field for amino acids, since the peptide did not have any chemical modifications. In the derivation of partial charges in the PMO part of the PPMO structure carried out in our previous study [1], we employed the Restrained Electro Static Potential (PESP) charge fitting procedure [2], implemented in the RED server (RESP ESP charge Derive Server) [3]. We utilized the RESP method in conjunction with HF theory and the 6-31G* basis set. Several *ab initio* calculations were carried out for each fragment, including the morpholino ring (5′-, 3′-, and central) with guanine, cytosine, thymine, and adenine bases, and with the MTEG linker. For each fragment we carried out 3 runs (total of 39 runs) over which the final partial charges were averaged. The Antechamber package [4] was employed to assign the all-atom types for atoms in these fragments. Molecular mechanical parameters were obtained from the bsc0$_{\chi OL3}$ force field for nucleic acids and from the ff14SB force field for proteins, for the included atom types. These include the equilibrium bond distances (r_0), spring constants for covalent bonds (k_b), equilibrium bond angles (θ_0), and spring constants for bending of bond angles (k_θ), as well as torsional angle parameters (magnitude associated with torsion energy ($V_n/2$), phase offset (γ) and periodicity (n)). For the atom types not covered in the bsc0$_{\chi OL3}$ and ff14SB force fields, the molecular mechanical parameters were generated using the general Amber force field GAFF [5]. For model detail of the force field development procedure, including the molecular mechanics parameters and partial charges, the reader should consult our previous work [1] (see also the SI in Ref. [1]). For each PPMO molecule, we created the topology files for each of the "morpholino nucleotides", for the MTEG linker, and for the peptide part. Next, we linked "morpholino nucleotides" through the phosphorodiamidate groups, added the MTEG linker at the 5′-end, and linked the poly-Arg peptide to the 3′-end of each of the target sequences (see Fig. 1 in the main part). These structures were used in all the MD simulations described in the main part.

Reference structures for calculation of thermodynamic state functions: To generate the unfolded reference structures for the 22-mer, 25-mer, and 30-mer PPMO molecules, we performed the all-atom MD simulations of thermal unfolding using the Generalized Born (GB) model of implicit solvation [6] implemented in pmemd [7]. We used the solution conformations obtained from the numerical fit of the theoretical CD profiles to the experimental CD curves. Each of the PPMO molecules were gradually heated from 300 K to 500 K over a 2.5-μs time interval to unfold (see Fig. S2). Next, the Support Vector Classifier was used to identify the 'unfolded' conformations (Fig. S2). We selected a total of 62, 98, and 84 extended conformations for the 22-mer, 25-mer, and 30-mer PPMO, respectively. Using these structures, the average reference enthalpy (H_{ref}) and reference entropy (S_{ref}) were then generated for the extended initial conformations for the 22-mer, 25-mer, and 30-mer PPMOs displayed in Fig. 1B in the main part.

Classification of folded and unfolded conformations of PPMOs: We applied the Support Vector Machines (SVM) to perform classification of the 22-mer, 25-mer, and 30-mer PPMO conformations into

the folded and unfolded conformation classes. We employed We carried out 4 independent 2.5-μs long thermal unfolding MD simulations to find the unfolded conformations for the 22-mer, 25-mer, and 30-mer PPMO molecules. The first 4 most populated conformations for each PPMO were used as initial structures (first 5 of them are shown in Fig. 2 in the main part). During these simulations, each PPMO was gradually heated from 300 K to 500 K. The numerical output (coordinate and energy files) was then used to identify the most representative molecular properties that show the unfolding transitions in the 22-mer, 25-mer, and 30-mer PPMO molecules at high temperatures. *Order parameters:* The temperature-dependent profiles of the end-to-end distance X, radius of gyration R_g, number of base stacks N_{BS}, and Solvent Accessible Surface Area $SASA$ showed the sigmoidal shape characteristic of a phase transition (Fig. S2). This enabled us to select the unfolded conformations of the 22-mer, 25-mer, and 30-mer PPMOs in order to form the training set for the Machine Learning based classification of the ensemble of conformations for these PPMOs. We selected the conformations for which the values of X, R_g, N_{BS}, and $SASA$ fall within the upper 90% of their maximum values, as indicated by the horizontal dashed lines in Figs. S2A-B.

For each PPMO system, we constructed the dataset with the values of X, R_g, N_{BS}, $SASA$ and $eRMSD$. For a manifold of various conformations of the PPMO molecules, obtained from the equilibrium MD simulations in an aqueous solution at 300 K, we constructed datasets $D_{eq,22}$, $D_{eq,25}$ and $D_{eq,30}$ for the 22-mer, 25-mer, and 30-mer PPMOs, respectively. The numerical output data for PPMOs' conformations, obtained from the MD simulations of thermal unfolding, were used to construct datasets $D_{unf,22}$, $D_{unf,25}$ and $D_{unf,30}$ for the 22-mer, 25-mer, and 30-mer PPMOs, respectively. For each PPMO system, these datasets were combined into datasets D_{22}, D_{25} and D_{30}, with two classes labeled 'Folded' and 'Unfolded'. These combined datasets were randomly separated into two halves forming the training sets ($D_{train,22}$, $D_{train,25}$ and $D_{train,30}$) and the test sets ($D_{test,22}$, $D_{test,25}$ and $D_{test,30}$). Next, we employed SVM to perform data classification, i.e. separation of PPMOs' structures into the folded conformations ('Folded' class) and the unfolded conformations ('Unfolded' class). The implementation of the SVM algorithm was based on the Python scikit-learn package [8]. Since the test sets contain only one half of the unfolded structures generated at 300 K, next, we applied these pre-trained SVM models to the data sets $D_{eq,22}$, $D_{eq,25}$ and $D_{eq,30}$, in order to extract all the unfolded PPMOs' conformations that would exclude conformations from the MD simulations of thermal unfolding. Therefore, we identified 62 conformations for the 22-mer PPMO, 98 conformations for the 25-mer PPMO, and 84 conformations for the 30-mer PPMO. The classification results for the 30-mer PPMO conformations into the 'Folded' class and 'Unfolded' class are displayed in Fig. S2 showing two- and three-dimensional projections of the separating hypersurface, i.e. N_{BS} vs. R_g (Fig. S2C) and N_{BS} vs. $SASA$ (Fig. S2D) and X vs. R_g vs. $SASA$ (Fig. S2E).

Supplemental Table

Table S1. Molecular properties and energetic characteristics of solution conformations of 22-mer PPMO, 25-mer PPMO, 30-mer PPMO: Shown for each principal solution conformer i = I-III, which account for ~55-70% of the ensemble populations, are the equilibrium population w_i, end-to-end distance X_i, radius of gyration $R_{g,i}$, number of base pairs $N_{BP,i}$, numbers of base stacks $N_{BS,i}$, solvent accessible surface area ($SASA_i$), and intrinsic viscosity η_i. Also shown are changes in free energy ΔG_i and enthalpy ΔH_i for folding of PPMOs (at $T = 300$ K). Data for X_i, $R_{g,i}$, $SASA_i$, ΔG_i, and ΔH_i for PPMOs (bolded text) are compared with the contributions to these quantities from the PMO part (separated by the slash).

**22-mer PPMO** / PMO in PPMO								
	w_i	X_i, nm	$R_{g,i}$, nm	$N_{BP,i}$/ $N_{BS,i}$	$SASA_i$, Å^2	η_i, cm^3/g	ΔG_i, kcal/mol	ΔH_i, kcal/mol
I	**0.26**	**1.9**/1.0	**1.3**/1.3	**9/4**	**4791**/3613	**5.3**	**-58**/-45	**-93**/-87
II	**0.17**	**1.7**/1.7	**1.4**/1.4	**7/5**	**5314**/3967	**4.5**	**-42**/-23	**-57**/-52
III	**0.09**	**3.2**/3.2	**1.4**/1.4	**2/5**	**5110**/3855	**4.5**	**-57**/-16	**-82**/-46
**25-mer PPMO** / PMO in PPMO								
	w_i	X_i, Nm	$R_{g,i}$, Nm	$N_{BP,i}$/ $N_{BS,i}$	$SASA_i$, Å^2	η_i, cm^3/g	ΔG, kcal/mol	ΔH, kcal/mol
I	**0.30**	**1.3**/1.7	**1.5**/1.4	**5/8**	**5215**/4632	**6.3**	**-83**/-72	**-118**/-90
II	**0.25**	**1.6**/1.8	**1.4**/1.4	**5/10**	**5763**/4820	**5.0**	**-65**/-58	**-97**/-77
III	**0.12**	**2.3**/1.8	**1.3**/1.3	**2/7**	**5486**/4911	**4.2**	**-79**/-48	**-101**/-66
**30-mer PPMO** / PMO in PPMO								
	w_i	X_i, nm	$R_{g,i}$, Nm	$N_{BP,i}$/ $N_{BS,i}$	$SASA_i$, Å^2	η_i, cm^3/g	ΔG, kcal/mol	ΔH, kcal/mol
I	**0.27**	**2.3**/1.3	**1.5**/1.5	**5/10**	**6506**/5596	**6.1**	**-84**/-44	**-104**/-71
II	**0.23**	**2.2**/2.5	**1.4**/1.4	**6/12**	**6179**/5057	**4.3**	**-80**/-37	**-114**/-68
III	**0.22**	**2.4**/3.4	**1.4**/1.4	**3/10**	**6276**/5406	**4.7**	**-60**/-18	**-110**/-52

Table S2. Molecular properties and thermodynamic state functions for the three therapeutic 22-mer, 25-mer, and 30-mer PPMOs: Presented are the statistics (averages and standard deviations) of the radius of gyration R_g, number of base pairs N_{BP}, number of base stackings N_{BS}, solvent accessible surface area $SASA$, intrinsic viscosity $[\eta]$, and Huggins constant k_H. Also shown are the changes in free energy ΔG and enthalpy ΔH for folding for the average PPMO solution structures (at $T = 300$ K); these quantities were calculated using the PPMOs' unfolded structures as the reference states (Fig. 1B) and the 6-7 most populated structures. Data for the PPMO structures are compared with data for the PMO structures from our previous study [1] (separated by the slash). The largest and the smallest parameter values are given in red and light blue color.

Parameter	22-mer PPMO / 22-mer PMO	25-mer PPMO / 25-mer PMO	30-mer PPMO / 30-mer PMO
R_g, nm	1.5±0.2 / 1.5±0.2	1.4±0.1 / 1.4±0.1	1.5±0.2 / 1.7±0.1
N_{BP}	6.0±2.1 / 3.3±0.9	4.9±2.9 / 5.9±1.1	4.5±2.2 / 6.4±4.2
N_{BS}	5.1±1.7 / 6.5±3.6	8.4±2.8 / 8.3±3.2	10.9±2.4 / 8.7±3.0
$SASA$, Å^2	5247±321 / 4538±248	5339±339 / 4819±158	6555±361 / 5945±341
$[\eta]$, cm^3/g	4.5±0.6 / 4.5±0.6	4.6±0.5 / 4.7±0.6	6.1±0.6 / 6.2±0.8
k_H	4.5 / 4.5	9.7 / 9.9	3.7 / 3.8
ΔG, kcal/mol	-49±9 / -34±15	-71±11 / -51±15	-70±23 / -50±10
ΔH, kcal/mol	-86±21 / -57±31	-103±22 / -89±19	-103±37 / -103±21

Supplemental Figures

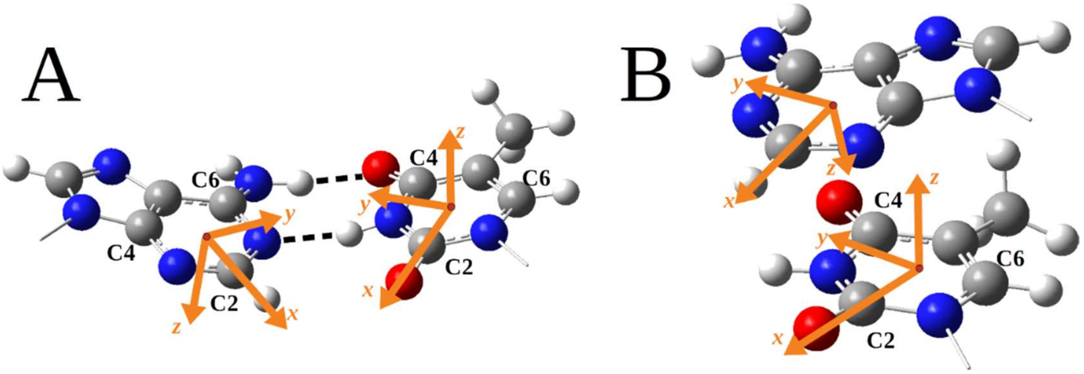

Figure S1. Base pairing and base stacking for PPMO molecules: Panel **A**: Local coordinate systems for purines and pyrimidines used in the calculation of base pairing and base stacking interactions (see Supplemental Methods). The center of the base ring atoms C2, C4, and C6 represents the origin of the coordinate system as shown in the graph. The x-and y-axes lie in the plane of the base while the z-axis is normal to the xy-plane. The x-axis is pointed in the C2-atom direction, and the y-axis is pointed toward the C4-atom (for C and U) or toward the C6-atom (for A and G). Two bases are forming a base pair via hydrogen bonds shown as dashed black lines. Panel **B**: The same x-, y- and z-axes from Panel **A** were used to describe the formation of base stacking interaction.

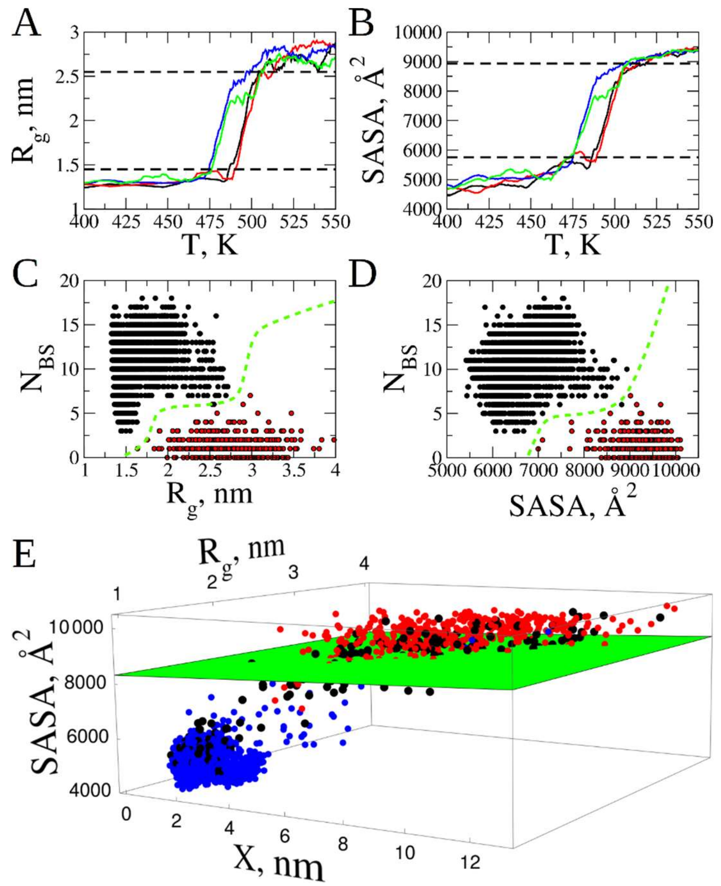

Figure S2. Dynamic structural properties associated with thermal unfolding of 30-mer PPMO: Panels **A-B** display temperature-dependent profiles of the secondary and tertiary structural properties of the 30-mer from three independent 2.5-μs MD simulations of gradual heating of this PPMO from 300 K to 500 K: radius of gyration R_g (panel **A**) and $SASA$ (panel **B**). Only the results from the 400 K – 500 K portion of the thermal unfolding experiments are shown. Horizontal dashed-dotted lines separate the regions corresponding to the folded and unfolded conformations of PPMOs. Also shown are the 2D scatter plots of a number of base-stacking interactions N_{BS} vs. R_g (panel **C**) and vs. $SASA$ (panel **D**) for the 30-mer PPMO, and 3D scatter plot of end-to-end distance X vs. R_g vs. $SASA$ (panel **E**). The black data points correspond to the 30-mer PPMO's conformations extracted from the equilibrium MD simulations at 300 K, while the red data points represent the conformations observed in the simulations of thermal unfolding, which were classified as 'Unfolded', and blue data points represent the conformations observed in the MD simulations of thermal unfolding, which were classified as 'Folded' (panel **E** only). In panels **C**, **D**, and **E**, the green dashed lines (plane) represent the two-dimensional (three-dimensional) projection of an optimal hypersurface obtained using the Support Vector Classifier to separate the conformations that belong to the 'Folded' class and 'Unfolded' class. In panels **C** and **D**, the black data points under the decision boundary line, which belong to the 'Unfolded' class, are the initial unfolded (reference) structures used in data analysis (see Fig. 1B in the main text). In panel **E**, the black data points above the hyperplane, which belong to the 'Unfolded' class, are also the initial unfolded (reference) structures used in data analysis (see Fig. 1B in the main text).

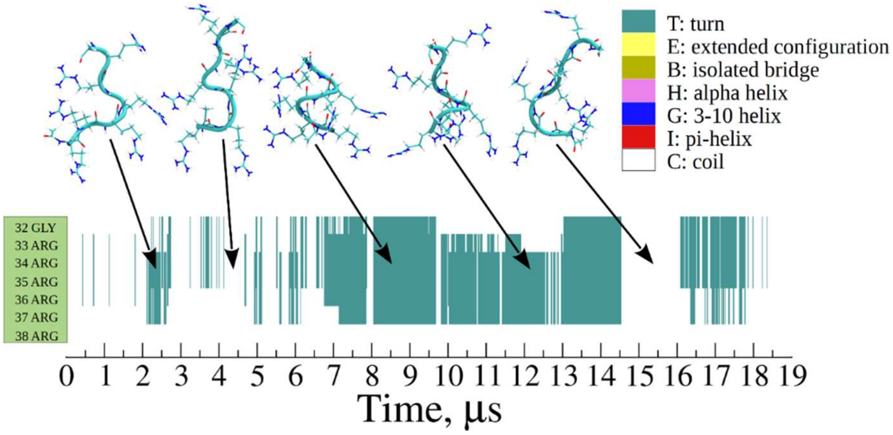

Figure S3. Secondary structure propensity for the peptide portion of the 22-mer, 25-mer, and 30-mer PPMOs: Displayed are the dynamic changes in the secondary structure for the peptide portion in the 30-mer PPMO from the 19-μs Molecular Dynamics simulations (all 15 trajectories combined in one long run). Color denotation for various secondary structure elements (i.e. α-helix, 3_{10}-helix, π-helix, β-strand, random coil, extended state, turn, and bridge) is shown in the graph. Also shown are several structure snapshots corresponding to 5 timepoints: 2.5 μs, 4.5 μs, 8.6 μs, 12.2 μs, and 15.5 μs. Only random coil and turn are observed during 19 μs of conformational dynamics (similar results were obtained for the 22-mer and 25-mer PPMOs).

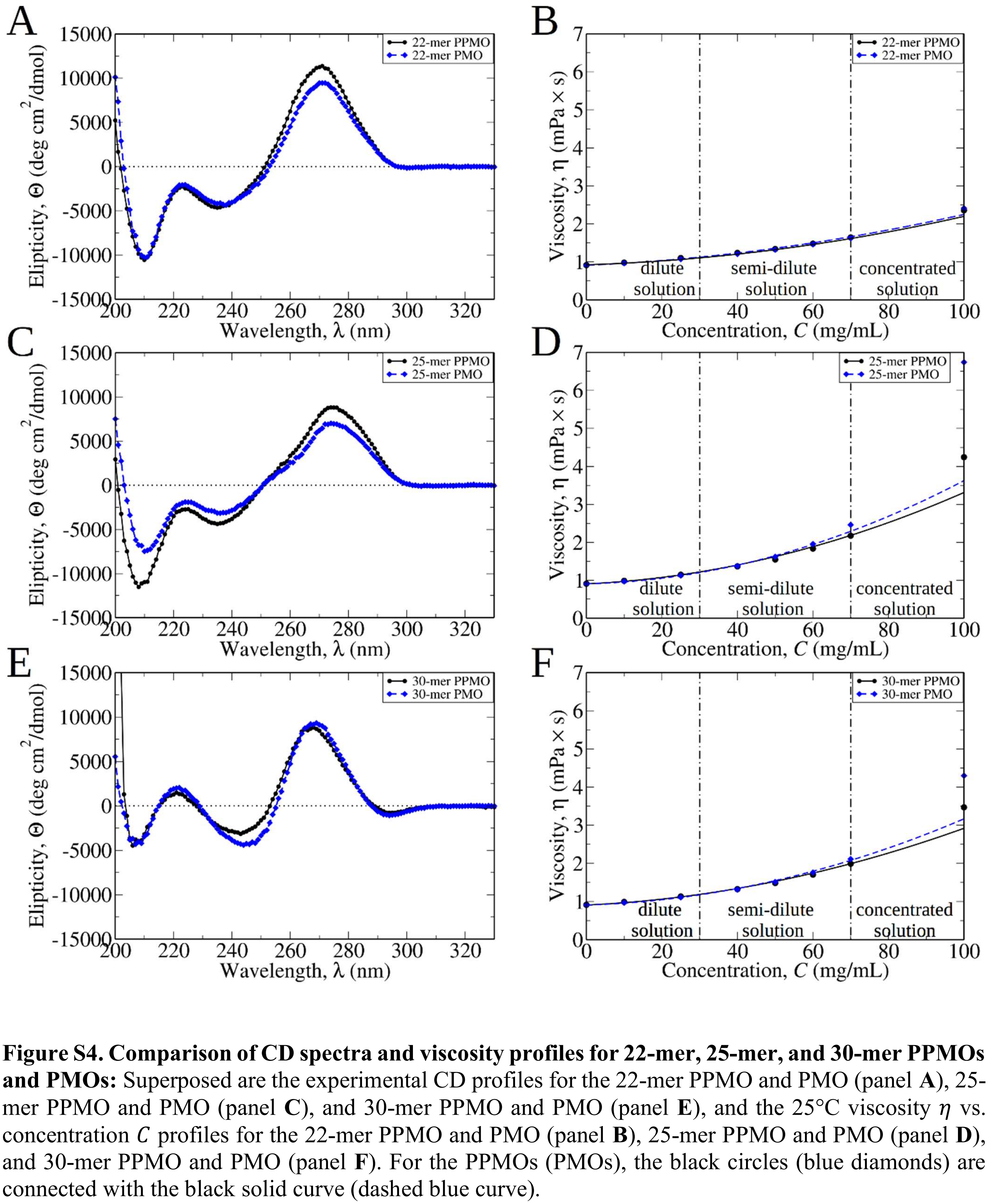

Figure S4. Comparison of CD spectra and viscosity profiles for 22-mer, 25-mer, and 30-mer PPMOs and PMOs: Superposed are the experimental CD profiles for the 22-mer PPMO and PMO (panel **A**), 25-mer PPMO and PMO (panel **C**), and 30-mer PPMO and PMO (panel **E**), and the 25°C viscosity η vs. concentration C profiles for the 22-mer PPMO and PMO (panel **B**), 25-mer PPMO and PMO (panel **D**), and 30-mer PPMO and PMO (panel **F**). For the PPMOs (PMOs), the black circles (blue diamonds) are connected with the black solid curve (dashed blue curve).

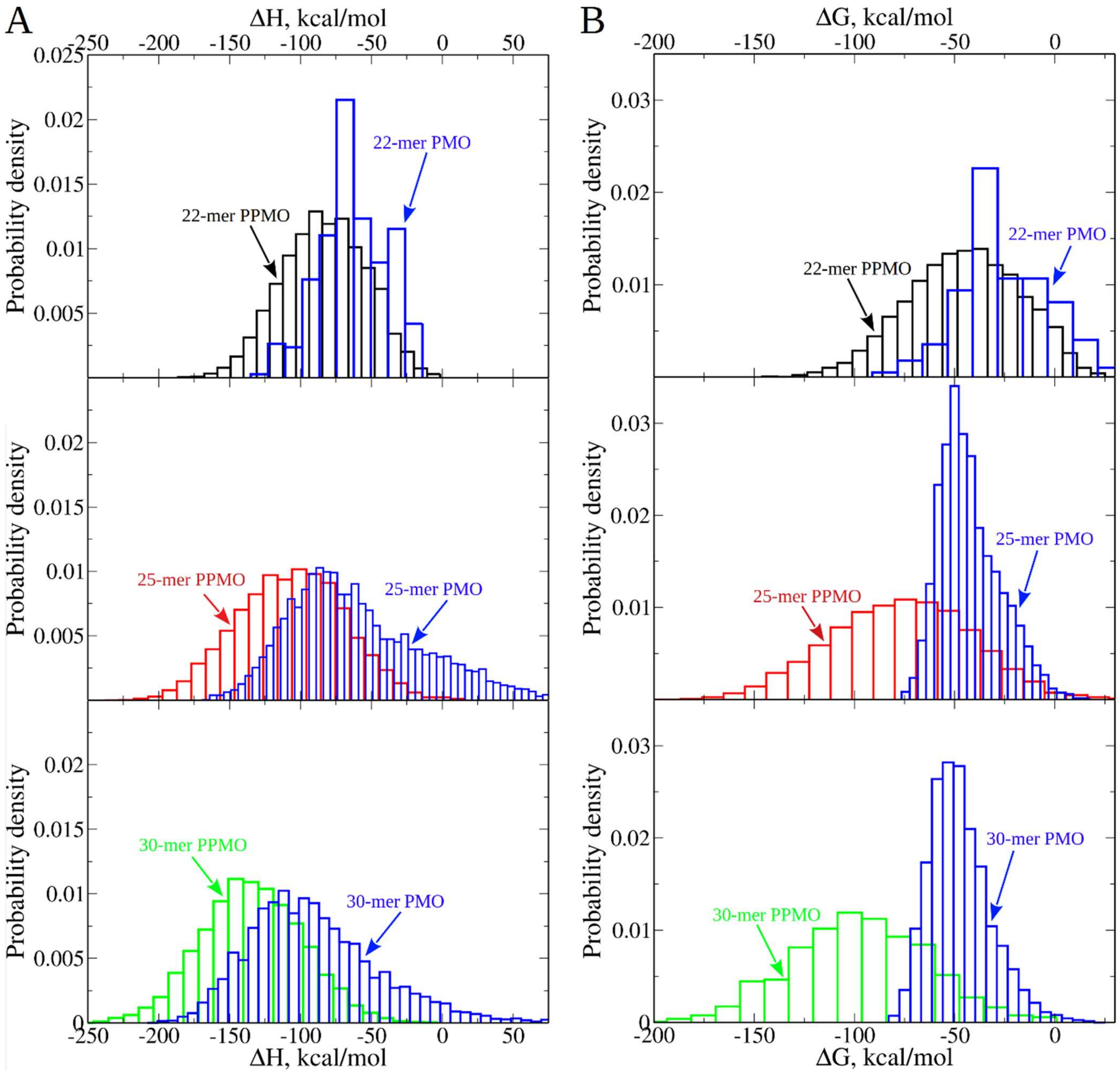

Figure S5. Histograms of thermodynamic state functions for 22-mer, 25-mer, and 30-mer PMOs and PPMOs: Shown are the histogram-based estimates of the normalized probability density functions (probability distributions) for the enthalpy change ΔH (panel **A**) and free energy change ΔG (panel **B**) for folding of the 22-mer, 25-mer, and 30-mer PPMO (black, red, and green bars, respectively) and for folding of the 22-mer, 25-mer, and 30-mer PMO (blue bars) [9]. The probability distributions for the PPMOs were sampled using the output from the equilibrium MD simulations at $T = 300$ K temperature (see Materials and Methods in the main text). The probability distributions for PMOs were taken from our previous study [1].

Supplemental Movies

Video S1. Folding of the 25-mer PPMO: The movie shows the conformational transition in the 25-mer PPMO from the extended state to the collapsed state (folded state) as observed in a 190-ns MD simulation run at $T = 300$ K. The MD run was carried out in explicit water (cyan transparent spheres). The PPMO molecule is shown in the Licorice representation (sticks) and in the Twister representation for the PMO backbone (blue line). Peptide portion of PPMO is shown in cyan color. The MTEG -linker is shown in orange, A and T bases are shown in green, and C and G bases are shown in red. The length of the movie is 26 s (the movie is played ~137,000,000 times slower than the computational experiment).

Video S2. Conformational dynamics of the 25-mer PMO: The movie shows conformational fluctuations of the 25-mer in the folded state as observed in a 3-μs MD simulation run at $T = 300$ K. The MD run was carried out in explicit water (cyan transparent spheres). The PPMO molecule is shown in the Licorice representation (sticks) and in the Twister representation for the PMO backbone (blue line). Peptide portion of PPMO is shown in cyan color. The MTEG -linker is shown in orange, A and T bases are shown in green, and C and G bases are shown in red. The length of the movie is 44 s (the movie is played ~15,000,000 times slower than the computational experiment).

Supplemental references:

1. Maksudov, F., Kliuchnikov, E., Pierson, D., Ujwal, M.L., Marx, K.A., Chanda, A., Barsegov, V. Therapeutic phosphorodiamidate morpholino oligonucleotides: Physical properties, solution structures, and folding thermodynamics. *Mol Ther Acids*. 2023;31:631-647.
2. Cieplak, P., Cornell, W.D., Bayly, C., Kollman, P.A. Application of the multimolecule and multiconformational RESP methodology to biopolymers: Charge derivation for DNA, RNA, and proteins. *J Comput Chem*. 1995;16(11):1357-1377.
3. Vanquelef, E., Simon, S., Marquant, G., Garcia, E., Klimerak, G., Delepine, J.C., Cieplak, P., Dupradeau, F.Y. RED Server: a web service for deriving RESP and ESP charges and building force field libraries for new molecules and molecular fragments. *Nucleic Acids Res*. 2011;39(suppl_2):W511-W517.
4. Wang, J., Wang, W., Kollman, P.A., Case, D.A. Automatic atom type and bond type perception in molecular mechanical calculations. *J Mol Graph Model*. 2006;25(2):247-260.
5. Wang, J., Wolf, R.M., Caldwell, J.W., Kollman, P.A., Case, D.A. Development and testing of a general amber force field. *J Comput Chem*. 2004;25(9):1157-1174.
6. Qiu, D., Shenkin, P.S., Hollinger, F.P., Still, W.C. The GB/SA continuum model for solvation. A fast analytical method for the calculation of approximate Born radii. *J Phys Chem A*. 1997;101(16):3005-3014.

7. Darden, T., York, D., Pedersen, L. Particle mesh Ewald: An N· log (N) method for Ewald sums in large systems. *J Chem Phys*. 1993;98(12):10089-10092.

8. Pedregosa, F., Varoquaux, G., Gramfort, A., Michel, V., Thirion, B., Grisel, O., Blondel, M., Prettenhofer, P., Weiss, R., Dubourg, V., et al. Scikit-learn: Machine learning in Python. *J Mach Learn Res*. 2011;12:2825-2830.

9. Bura, E., Zhmurov, A., Barsegov, V. Nonparametric density estimation and optimal bandwidth selection for protein unfolding and unbinding data. *J Chem Phys*. 2009;130(1):015102.
